# Synthesis and in vitro biochemical evaluation of oxime bond-linked daunorubicin–GnRH-III conjugates developed for targeted drug delivery

**DOI:** 10.3762/bjoc.14.64

**Published:** 2018-04-04

**Authors:** Sabine Schuster, Beáta Biri-Kovács, Bálint Szeder, Viktor Farkas, László Buday, Zsuzsanna Szabó, Gábor Halmos, Gábor Mező

**Affiliations:** 1 MTA-ELTE Research Group of Peptide Chemistry, Hungarian Academy of Sciences, Eötvös L. University, 1117 Budapest, Hungary; 2 Institute of Chemistry, Eötvös L. University, 1117 Budapest, Hungary; 3 Research Centre for Natural Sciences, Institute of Enzymology, Hungarian Academy of Sciences, 1117 Budapest, Hungary; 4 MTA-ELTE Protein Modelling Research Group, Hungarian Academy of Sciences, Eötvös L. University, 1117 Budapest, Hungary; 5 Department of Biopharmacy, Faculty of Pharmacy, University of Debrecen, 4032 Debrecen, Hungary

**Keywords:** cytostatic effect, daunorubicin, drug-targeting, GnRH derivatives, oxime linkage

## Abstract

Gonadotropin releasing hormone-III (GnRH-III), a native isoform of the human GnRH isolated from sea lamprey, specifically binds to GnRH receptors on cancer cells enabling its application as targeting moieties for anticancer drugs. Recently, we reported on the identification of a novel daunorubicin–GnRH-III conjugate (GnRH-III–[^4^Lys(Bu), ^8^Lys(Dau=Aoa)] with efficient in vitro and in vivo antitumor activity. To get a deeper insight into the mechanism of action of our lead compound, the cellular uptake was followed by confocal laser scanning microscopy. Hereby, the drug daunorubicin could be visualized in different subcellular compartments by following the localization of the drug in a time-dependent manner. Colocalization studies were carried out to prove the presence of the drug in lysosomes (early stage) and on its site of action (nuclei after 10 min). Additional flow cytometry studies demonstrated that the cellular uptake of the bioconjugate was inhibited in the presence of the competitive ligand triptorelin indicating a receptor-mediated pathway. For comparative purpose, six novel daunorubicin–GnRH-III bioconjugates have been synthesized and biochemically characterized in which ^6^Asp was replaced by D-Asp, D-Glu and D-Trp. In addition to the analysis of the in vitro cytostatic effect and cellular uptake, receptor binding studies with ^125^I-triptorelin as radiotracer and degradation of the GnRH-III conjugates in the presence of rat liver lysosomal homogenate have been performed. All derivatives showed high binding affinities to GnRH receptors and displayed in vitro cytostatic effects on HT-29 and MCF-7 cancer cells with IC_50_ values in a low micromolar range. Moreover, we found that the release of the active drug metabolite and the cellular uptake of the bioconjugates were strongly affected by the amino acid exchange which in turn had an impact on the antitumor activity of the bioconjugates.

## Introduction

Cancer is one of the most serious diseases worldwide and malignant tumors and metastases often lead to high mortality. Chemotherapy is a widely used method to treat cancerous diseases, but the lack of selectivity, drug-specific side-effects and toxicity to healthy tissues result in various complications, which restrict the application of chemotherapeutics. A promising treatment option to overcome these drawbacks can be targeted tumor therapy. This approach is based on the fact that receptors for many regulatory ligands such as peptide hormones are overexpressed on the surface of various cancer cells including gonadotropin-releasing hormone receptors (GnRH-R) [1]. Therefore, these peptides are suitable for specific drug targeting to tumor cells. The native ligand of this receptor is GnRH-I (<EHWSYGLRPG-NH_2_, where <E is pyroglutamic acid) which is synthesized and released within the hypothalamus. GnRH stimulates the synthesis and release of the regulatory pituitary glycoprotein luteinizing hormone (LH) and follicle stimulating hormone (FSH) which act on the gonads and regulate the production of the sex steroids androgen and estrogen [2].

In the last decades a large number of synthetic GnRH-I-analogues has been designed with the purpose to interact with the receptor and influence the release of pituitary gonadotropins LH and FSH [1,3–6]. The replacement of ^6^Gly by D-amino acids in human GnRH-I provides superagonists like the GnRH-I derivatives buserelin [^6^D-Ser(*t*-Bu), ^9^Pro-EA], goserelin [^6^D-Ser(*t*-Bu), ^10^Azagly-NH_2_], leuprolide [^6^D-Leu, ^9^Pro-EA] and triporelin [^6^D-Trp], which are used as pharmaceutical peptides to treat inter alia hormone dependent prostate and/or breast cancer [7].

Since the mid-1980s cytotoxic GnRH-I derivatives were developed and investigated to treat tumor cells [4–5,8–9]. Anthracyclines such as doxorubicin (Dox), daunorubicin (Dau) or epirubicin are frequently used anticancer drugs. Their mode of action is based on a planar ring system which is important for intercalation into DNA [10]. In this way, anthracyclines can affect a broad range of DNA processes leading to an inhibited synthesis of macromolecules such as mRNA and DNA [10–11]. More precisely, anthracyclines act as topoisomerase II toxins inhibiting DNA transcription and replication. They stabilize a DNA topoisomerase-II intermediate in which the DNA strands are separated and a specific tyrosine residue of the topoisomerase II is covalently linked to the DNA by formation of a tyrosine phosphodiester [10–12]. Moreover, anthracyclines provide a beneficial auto-fluorescence allowing the performance of fluorescence based studies like confocal laser scanning microscopy (CLSM) and fluorescence-activated cell sorting (FACS) to investigate the cellular uptake and the subcellular localization of the drug or the drug bioconjugates [13–14]. It is well known from the literature that anthracyclines accumulate in the nucleus, in that manner they also act as DNA stains [14–15].

The first cytotoxic GnRH-I derivative, which was investigated in preclinical and clinical studies, was zoptarelin-doxorubicin also known as AEZS-108 (previously AN-152) [16]. The anthracycline doxorubicin was conjugated to the ε-amino group of GnRH-I-[^6^D-Lys] by insertion of a glutaric acid linker. The resulting ester bond can be cleaved by carboxylesterases, leading to the release of the cytotoxic agent within the tumor cell. During clinical trials, only mild side effects were observed which are caused by premature drug release [17]. The receptor mediated uptake of zoptarelin has been investigated by blockage of GnRH receptors using an excess of the GnRH-I superagonist triptorelin [14]. In addition, the internalization and the intracellular localization of AN-152 were visualized by CLSM [14]. Despite all these promising findings, zoptarelin-doxorubicin did not achieve its primary endpoint in phase 3 clinical studies on endometrial cancer [18].

A natural isoform of the human GnRH-I, the sea lamprey analogue GnRH-III (<EHWSHDWKPG-NH_2_), was identified and characterized by Sower et al. [19]. Due to the significantly lower endocrine effect compared to GnRH-I and the specific binding to GnRH-Rs on cancer cells, GnRH-III might have advantage as a carrier for cytotoxic drugs, especially in case of hormone-independent tumors [20]. Based on these findings, GnRH-III has been used as an efficient homing device for targeted tumor therapy [21–22]. Moreover, it was demonstrated that a modification of the side chain of ^8^Lys did not hinder the receptor binding or the antiproliferative activity. Furthermore, the absence of the free ε-amino group additionally reduced the endocrine effect [23–24]. Thus, the ^8^Lys can be utilized as conjugation site for cytotoxic agents like anthracyclines. In the past decade, a variety of different linkage systems has been carried out including ester or hydrazine bonds, cathepsin-B labile spacers and oxime bonds [21–22,25].

Due to its structural properties, Dau cannot be attached to the homing device by an ester bond like Dox, because of the absence of the primary hydroxy group in position C-14. However, the C-13 carbonyl group of Dox/Dau provides a suitable conjugation site and can be used for the formation of oximes. We have recently reported that Dau was efficiently linked to the ^8^Lys side-chain by incorporation of an aminooxyacetic acid (Aoa) moiety [21,25]. The formed oxime linkage is more stable under physiological conditions than the ester bond resulting in a longer half-life of the conjugate during circulation. Nevertheless, the drug is released within the cancer cell by lysosomal enzymes, especially by cathepsin B, which leads to various Dau containing metabolites [26]. In case of GnRH-III–[^8^Lys(Dau=Aoa)] conjugates the smallest Dau metabolite obtained by lysosomal degradation is H-Lys(Dau=Aoa)-OH, which is able to bind to DNA resulting in a cytotoxic effect. A variety of oxime bond containing GnRH-III drug conjugates have been designed in our research group and their in vitro cytotoxic effects on hormone dependent human breast adenocarcinoma cancer cells (MCF-7) and on hormone independent human colon carcinoma cells (HT-29) were analyzed [22,25,27–28]. Thereby, it has been exposed, that an exchange of ^4^Ser by ^4^Lys followed by acylation of the ε-amino group with short chain fatty acids (SCFA) improved the cellular uptake and the antitumor activity [29]. Moreover, these GnRH-III bioconjugates displayed an enhanced stability in the presence of gastrointestinal enzymes. The most potent and efficient bioconjugate which has been evaluated in in vitro cytostatic effect measurements on human breast cancer cells (MCF7) and human colon cancer cells (HT-29), is GnRH-III–[^4^Lys(Bu), ^8^Lys(Dau=Aoa)] (**K2**). Recent studies demonstrated that the butyrilation of the lysine in position 4 not only leads to an increased in vitro but also to an enhanced in vivo antitumor activity [29–30].

In order to achieve a better understanding of the mechanism of action, the internalization and the intracellular localization, our lead compound **K2** was studied and monitored by CLSM. Furthermore, the cellular uptake of **K2** was evaluated in competition with the GnRH-I superagonist triptorelin by flow cytometry indicating a receptor mediated pathway.

To gain further information about sequence–activity relationship of GnRH-III, we studied the impact of ^6^Asp on the efficiency of tumor targeting. Since it is known from the literature that an incorporation of D-amino acids (D-Aaa) in position 6 of GnRH-I and II peptides can lead to an improved receptor binding affinity and an enhanced antiproliferative activity without substantial effect on the endocrine activity [31–33], we developed six novel GnRH-III–Dau conjugates in which the ^6^Asp was replaced by D-Aaa. Here we report on the synthesis of GnRH-III bioconjugates containing D-Asp, D-Glu or D-Trp in position 6 and Ser or Lys(Bu) in position 4. Moreover, the novel GnRH-III–Dau conjugates were compared systematically with our lead compound **K2** in terms of in vitro cytostatic effect, receptor binding affinity, cellular uptake and lysosomal digestion in the presence of rat liver lysosomal homogenate.

## Results and Discussion

### Synthesis of oxime bond-linked GnRH-III–[^4^Ser/Lys(Bu), ^6^Aaa, ^8^Lys(Dau=Aoa)] bioconjugates

**Scheme 1:**
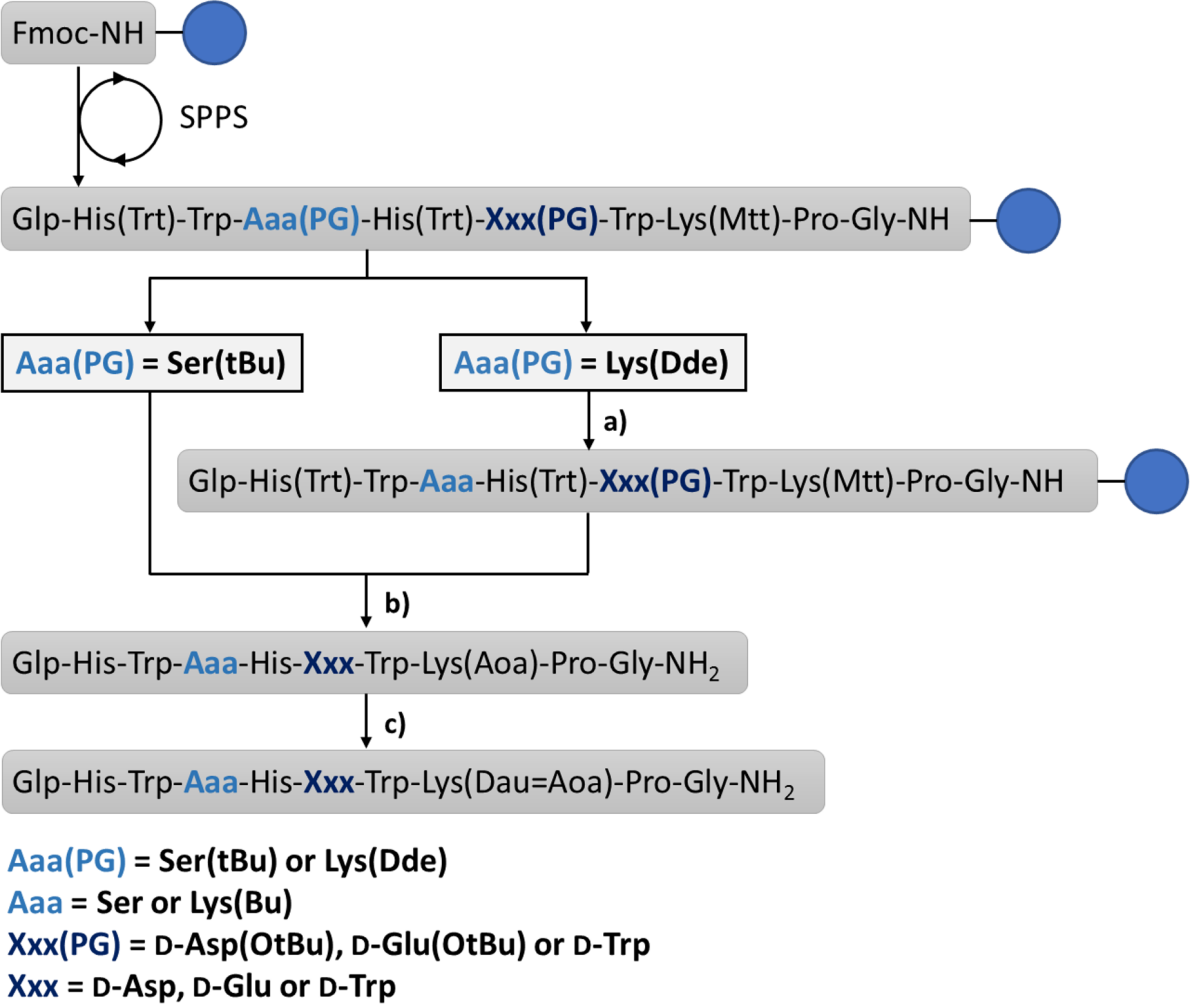
Syntheses of GnRH-III–[(^4^Lys(Bu)/^4^Ser, ^6^Aaa, ^8^Lys(Dau=Aoa)] bioconjugates. a) (1) 2% hydrazine in DMF 12 × 5 min; (2) 3 equiv butyric anhydride, 3 equiv DIPEA in DMF, 2 h. b) (1) 2% TFA in DCM, 6 × 5 min; (2) 10% DIPEA in DCM, 3 × 5 min; (3) 3 equiv Boc-Aoa-OH, 3 equiv HOBt, 3 equiv DIC in DMF, 1 h; (4) 95% TFA, 2.5% TIS, 2.5% H_2_O, 10 equiv H-Aoa-OH, 2h. c) 1.3 equiv Dau in 0.1 M NH_4_OAc buffer (pH 5), overnight. Fmoc: 9-fluorenylmethoxycarbonyl, SPPS: solid-phase peptide synthesis, Dde: 1-(4,4-dimethyl-2,6-dioxocyclohex-1-ylidene)ethyl, Mtt: 4-methyltrityl, Bu: butyryl, Aoa: aminooxyacetyl, Dau: daunorubicin, blue circle: Rink amide MBHA-resin.

The GnRH-III bioconjugates were prepared as shown in Scheme 1. All peptides were synthesized by standard Fmoc-SPPS using orthogonal lysine protecting groups. Fmoc-Lys(Dde)-OH was incorporated in position 4 and Fmoc-Lys(Mtt)-OH in position 8. After peptide chain elongation the Dde group was removed and ^4^Lys was butyrylated by using butyric anhydride. Afterwards, the Mtt group was cleaved under mild acidic conditions, followed by Boc-Aoa-OH coupling. After cleavage from the resin with an appropriate TFA–scavenger mixture and purification of the crude compounds by preparative HPLC, the attachment of daunorubicin via oxime linkage was carried out in solution as previously reported [21]. The resulting GnRH-III–Dau conjugates were purified by preparative HPLC, the final products **K1**, **K2** and **1**–**6** were characterized by analytical HPLC and mass spectrometry (Table 1, Supporting Information File 1, Figures S1–S8). The bioconjugates could be obtained in yields up to 27% over all synthesis and purification steps. The lower yields of the D-amino acid containing derivatives, especially in case of D-Trp are related mainly to their decreased solubility compared to the parent compounds. Moreover, the free aminooxy group is highly reactive towards aldehydes and ketones leading to the formation of unwanted side-products [34]. Aminooxy acetylated peptides prone to react with traces of acetone or formaldehyde (e.g., from the softeners of the plastic tubes) not only during the reaction steps (cleavage, ligation) but also under the HPLC purification conditions which has a high impact on the yield [35].

**Table 1:** Chemical characteristics of Dau–GnRH-III bioconjugates.

Code	[^8^Lys(Dau=Aoa)]–GnRH-III compound	Purity [%]	RP-HPLC *t*_R_ [min]^a^	ESIMS MW_cal_/MW_exp_ [g/mol]^b^	Yield [%]^c^

**K1**	[^6^L-Asp]	>97	27.8	1841.89/1841.66	22
**1**	[^6^D-Asp]	>96	28.0	1841.89/1841.60	8
**2**	[^6^D-Glu]	>98	29.2	1855.91/1855.70	14
**3**	[^6^D-Trp]	>95	32.5	1913.01/1912.80	7
**K2**	[^4^Lys(Bu),^6^L-Asp]	>97	29.3	1953.071952.79	27
**4**	[^4^Lys(Bu),^6^D-Asp]	>98	29.5	1953.07/1952.90	9
**5**	[^4^Lys(Bu),^6^D-Glu]	>96	29.7	1966.93/1966.70	7
**6**	[^4^Lys(Bu),^6^D-Trp]	>97	32.6	2024.03/2023.70	6

^a^Column: Phenomenex Luna C18 column (250 mm × 4.6 mm) with 5 µm silica (100 Å pore size); gradient: 0 min 0% B, 5 min 0% B, 50 min 90% B; eluents: 0.1% TFA in water (A) and 0.1% TFA in acetonitrile/water (80:20, v/v) (B); flow rate: 1 mL/min; detection at 220 nm. ^b^Bruker Daltonics Esquire 3000+ ion trap mass spectrometer. ^c^Yield over all synthetic and purification steps.

### Secondary structure determination by electronic circular dichroism spectroscopy

**Figure 1:**
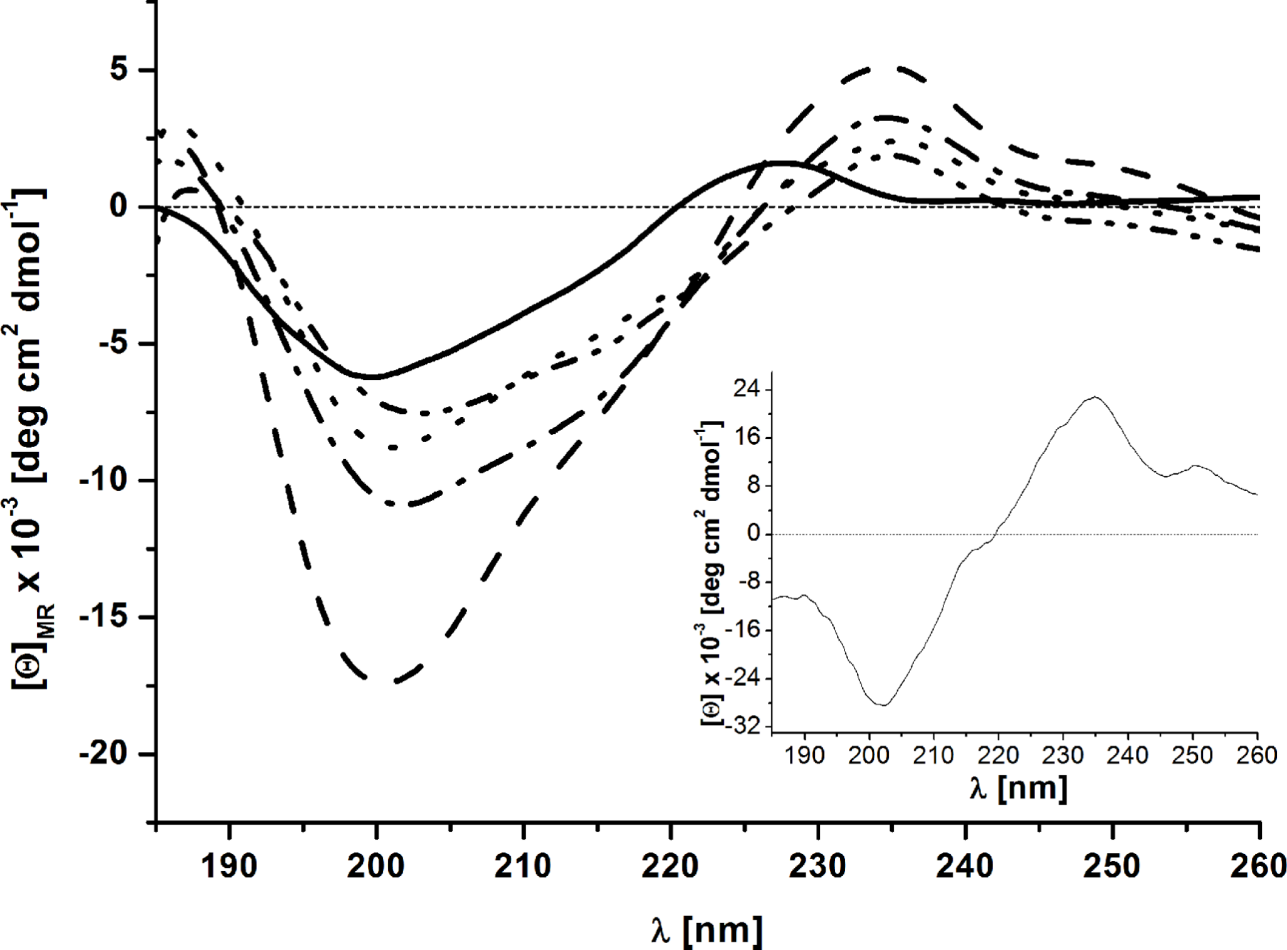
Far-UV ECD spectra of GnRH-III and its drug conjugates in water (GnRH-III solid, **K1** dash, **K2** dot, **1** dash dot, **4** dash dot dot). Insert: far-UV ECD spectra of daunorubicin.

ECD spectra of GnRH-III–[^4^Ser/^4^Lys(Bu), ^6^D/L-Asp, ^8^Lys(Dau=Aoa)] (**K1**, **K2**, **1** and **4**) were measured in aqueous solution to study the influence of the exchange of L-Asp to D-Asp in position 6 on the peptide conformation. The D-Trp containing analogs were not soluble under this condition, while we did not expect significant influence of ECD spectra from the exchange of D-Asp to D-Glu. The ECD spectra of all four GnRH-III peptide conjugates reveal two distinct bands: one negative at 200 nm and one positive at 235 nm (Figure 1, Supporting Information File 1, Figures S15 and S16). The negative band is also present in the GnRH-III peptide without the daunorubicin (Dau) part. This indicates that the main conformational preferences are not changed by the conjugation. The shapes of the ECD curves show a highly dynamic peptide structure in water. This is in agreement with the NMR study made by Pappa et al. that presented an extended and more flexible structure of GnRH-III than GnRH-I which has rather more defined U-shape structure [36]. In case of the positive band (235 nm) we can conclude that this is a contribution of the Dau part of the molecules because it has a positive band near 230 nm and in the conjugates this band is shifted to 235 nm. The Dau has also a negative band at 200 nm.

The only difference between the D-Asp derivatives and the original peptides is located near 215 nm. This sign indicates the presence of increased contribution of β-sheet like secondary structure in case of D-Asp derivatives. Because no aggregation could be observed in HPLC or MS measurements, we assume that the presence of the β-hairpin structure is a feasible conception in these cases.

### Stability/degradation of the GnRH-III bioconjugates

Drug delivery systems (DDS) are promising therapeutics for tumor therapy providing a selective application to tumor cells and a reduction of toxic side effects. To ensure these benefits, stability and degradation studies are of great importance during DDS development. In vitro degradation studies in the presence of gastrointestinal enzymes and lysosomal homogenate as well as stability analyses in human serum provide valuable information [37]. On the one hand toxic side effects caused by premature drug release should be avoided, on the other hand an intracellular drug release from the bioconjugates in the targeted cell is mandatory to assure the antitumor activity. Previously, we reported on the stability of the bioconjugates GnRH-III–[^8^Lys(Dau=Aoa)] (**K1**) and GnRH-III–[^4^Lys(Ac), ^8^Lys(Dau=Aoa)] in 90% human serum and in the presence of digestive enzymes trypsin and α-chymotrypsin [26,38]. It was found that both Dau–GnRH-III compounds were stable in human serum and trypsin at 37 °C for at least 24 h. Furthermore, the incorporation of an acetylated lysine instead of the native serine in position 4 decelerated the degradation by α-chymotrypsin which catalyzed the hydrolysis of the peptide bond exclusively between ^3^Trp and ^4^Aaa. A similar effect was observed for GnRH-III conjugates in which the ε-amino group of ^4^Lys was acylated by different SCFAs. It is worth to mention that the main cleavage sites of GnRH peptides by different enzymes are at both sides of serine in position 4 [39–40], that can be prohibited by replacement of Ser with an acylated lysine. Moreover, it has been shown that the stability is increased with the length of the side chain modification [29]. In this manner, the bioconjugate GnRH-III–[^4^Lys(Bu), ^8^Lys(Dau=Aoa)] (**K2**) displayed a two-fold higher stability in the presence of α-chymotrypsin than GnRH-III–[^4^Lys(Ac), ^8^Lys(Dau=Aoa)] [29,38].

The novel GnRH-III bioconjugates **1**–**6** have been analyzed for their stability/degradation in cell culture medium and in the presence of rat liver lysosomal homogenate. Reaction mixtures were incubated for 24 h at 37 °C, samples were taken at different time points and analyzed by analytical RP-HPLC and LC–MS. The approved bioconjugates **K1** and **K2** were analyzed in the same manner and the achieved results were used for a comparative evaluation of the novel GnRH-III–Dau conjugates.

The stability studies in cell culture medium revealed that all compounds were stable at 37 °C for 24 h which is in accordance with our previous results [29]. During this time, no decomposition of the conjugates was observed, demonstrating that no free drug or small drug containing metabolite was produced in medium during the treatment that might have influence on the in vitro biological assays.

Besides stability under physiological conditions, the release of the drug within cancer cells is of high relevance. In this study the anthracycline daunorubicin was used as anticancer agent. This drug interacts with DNA by intercalation and affects a broad range of DNA processing [11,41]. Orbán et al. recently reported that next to the free drug, also small Dau-containing metabolites revealed an antitumor activity [26,42].

**Figure 2:**
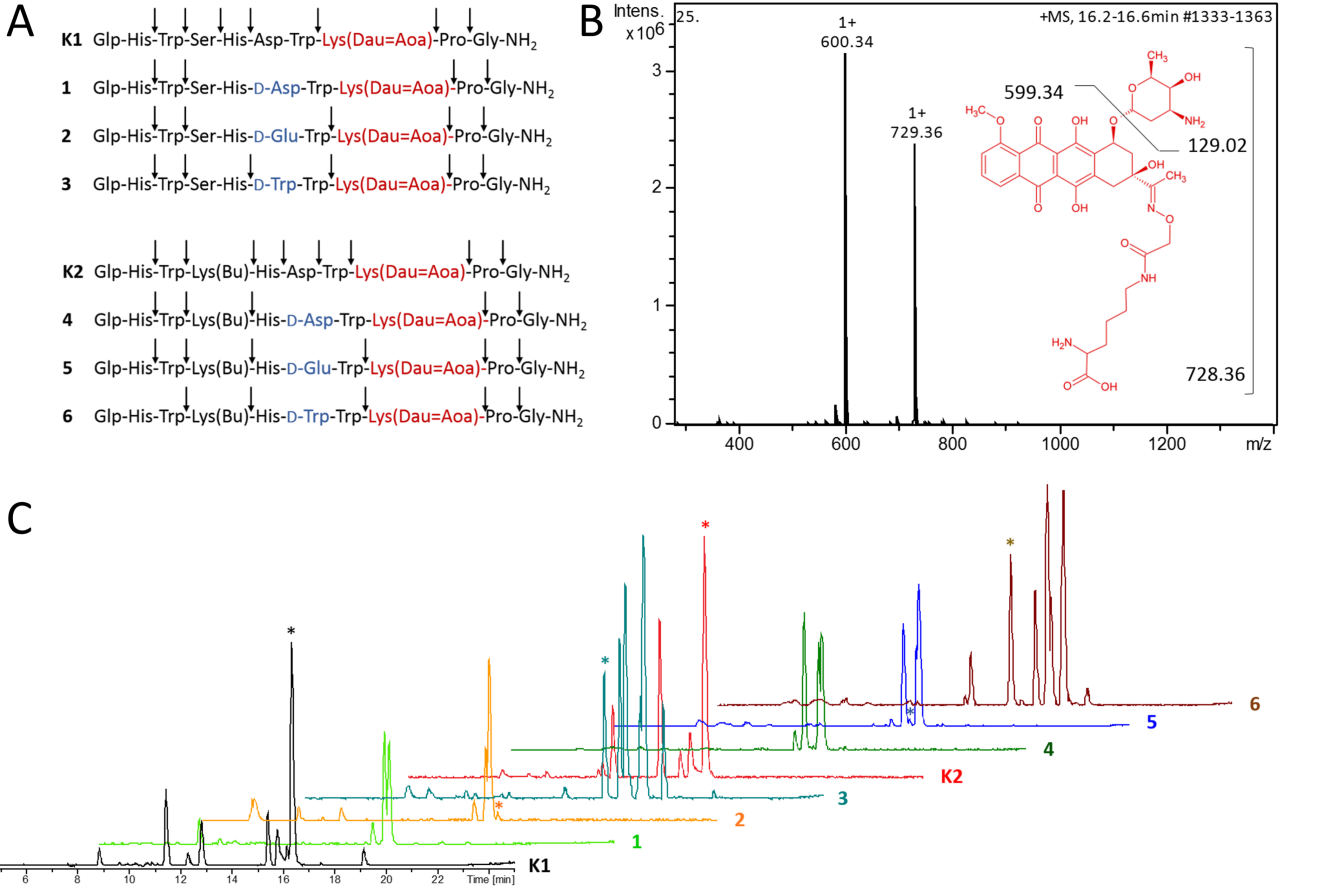
Degradation of the GnRH-III bioconjugates by rat liver lysosomal homogenate. A) Cleavage sites produced by the proteolysis of bioconjugates in the presence of rat liver lysosomal homogenate (full-line arrows). B) Structure of the smallest Dau-containing metabolite and its corresponding mass spectra (analysis of **K1** after 24 h incubation at the retention time 16.2–16.6 min of the LC–MS chromatogram). C) LC–MS chromatogram of the GnRH-III bioconjugates after 24 h of incubation with rat liver lysosomal homogenate at 37 °C (asterisk labeling peak of the smallest Dau-containing metabolite H-K(Dau=Aoa)-OH).

In order to gain insight into the cellular release of the drug, the degradation of the Dau–GnRH-III derivatives **1**–**6, K1** and **K2** was determined in the presence of rat liver lysosomal homogenate at 37 °C. As shown in Figure 2, all GnRH-III–Dau peptides were digested by lysosomal enzymes resulting in various peptide fragments (Supporting Information File 1, Table S1). However, the degradation level and the cleavage sites within the GnRH-III sequence differ considerably depending on the incorporated amino acids, whereby the hydrolyses of the *C*-terminus (H-Gly-NH_2_ and H-Pro-Gly-NH_2_) occurred first in all eight GnRH-III derivatives. Due to the high chemical and enzymatic stability of the oxime bond, no free Dau was detected by LC–MS. In accordance with our previous results, the digestion of the two peptides containing L-Asp in position 6 (**K1**, **K2**) provided the smallest Dau-containing metabolite (H-Lys(Dau=Aoa)-OH, *m/z* 729.36 [M + H]^+^). This metabolite could already be detected after 1 hour of incubation in case of **K2** and after 2 hours in case of **K1**. The ^6^D-Asp containing counterparts **1** and **4** exhibited higher lysosomal stabilities preventing the release of H-Lys(Dau=Aoa)-OH. On the contrary, the fragment H-Lys(Dau=Aoa)-OH could be identified in small amount after 24 h digestion of the bioconjugates **2** and **5** (^6^D-Glu). Surprisingly, in case of the ^6^D-Trp containing analogues **3**, **6**, the effective metabolite was delivered much faster (after 2 h **6** or 4 h **3**) and in a higher amount (highlighted peaks Figure 2C). Moreover, the detected fragment H-wWK(Dau=Aoa)-OH demonstrated that the D-Trp of conjugate **3** was accepted at the cleavage site of at least one lysosomal protease leading to improved release of the active metabolite. It might be assumed that D-Trp of GnRH-III–[^4^Lys(Bu), ^6^D-Tpr, ^8^Lys(Dau=Aoa)] (**6**) is also accepted at the cleavage site, but due to the prior hydrolysis of the ^7^Trp-^8^Lys(Dau=Aoa)-bond an evidential fragment has not been detected. A reason for these diversities might be the subsite specificities of the lysosomal proteases. For instance, lysosomal cysteine proteases also known as cathepsins show a broad substrate specificity [43]. Nearly all human cysteine proteases belong to the group of endopeptidases, whereby cathepsin B is also a carboxydipeptidase and cathepsin X displays carboxymono- and dipeptidase activity [44–46]. On the contrary, cathepsin C functions as an aminodipeptidase and cathepsin H reveals next to its endopeptidase activity also aminomonopeptidase activity [44,46]. Due to the variety of the detected fragments, we suggest that the rat liver homogenate contained a similar mixture of homolog cathepsins. For instance, the analyzed fragments of the ^6^L-Asp derivatives gave clear hints for the presence of endopeptidases, while the digestion of the ^6^D-Aaa compounds **1**–**3** gave only fragments which evidence the activity of exomono- and/or dipeptidases. Moreover, the obtained results for the bioconjugates that contain ^4^Lys(Bu) (**K2**, **4**–**6**) instead of serine indicate a proteolytic cleavage by lysosomal endopeptidases, which might be of great importance for the release of the smallest Dau-containing metabolite.

### Cytostatic effect of the bioconjugates

**Figure 3:**
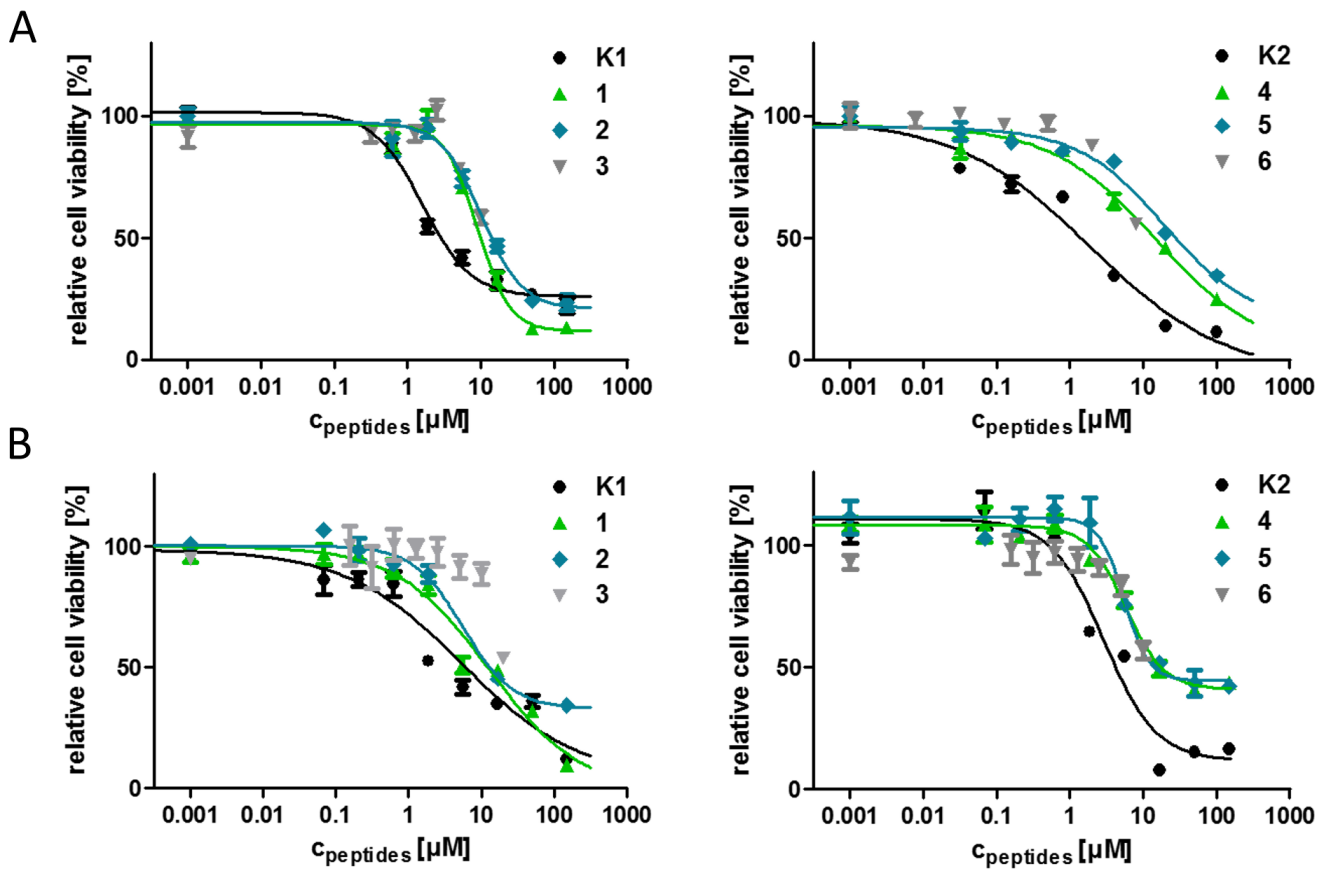
Cytostatic effect of the GnRH-III bioconjugates at different concentrations on A) HT-29 and B) MCF-7 cells after 24 h determined by alamarBlue^®^ cell viability assay. Experiments were carried out by using four replicates with *n* = 2, error bars represent standard deviation. Curves obtained by nonlinear regression (sigmoidal dose response). For IC_50_ values see Table 2.

Cell lines often function as the first model system of choice to study biological processes or to test the efficiency of drugs or drug conjugates and their cytotoxic effects. Immortal cell lines offer various benefits, for instance they are easy to handle, cost-effective and provide consistent sample and reproducible results [47]. Nevertheless, cell lines also present the disadvantage that after a period of continuous growth, cell characteristics can change and dedifferentiate in culture [47–48]. The serial passaging can cause genotypic and phenotypic variations and the state of confluency can also affect gene expression pattern in some cell lines [47,49]. Due to this, the usage of internal standards during the evaluation of new potential candidates might be necessary to ensure comparability with previous results. The cytostatic effect of the novel GnRH-III bioconjugates was determined on HT-29 human colon cancer and MCF-7 human breast cancer cell lines by alamarBlue^®^ assay. For a better comparison, the well-studied bioconjugates GnRH-III–[^8^Lys(Dau=Aoa)] (**K1**) and GnRH-III–[^4^Lys(Bu), ^8^Lys(Dau=Aoa)] (**K2**) were used as positive controls and internal standards. The corresponding IC_50_ values were calculated by using nonlinear regression (sigmoidal dose response) (Figure 3, Table 2). Unfortunately, the D-Trp containing compounds **3** and **6** started to precipitate in both cell culture media at higher concentrations limiting the concentration down to a maximum of 10 µM. This effect could not be prevented by using DMSO instead of ddH_2_O for the preparation of the stock solutions. Unfortunately, the lower concentration range with a maximum of 10 µM was not sufficient to achieve the dose-response. Nevertheless, both D-Trp-containing compounds provided a decreased cell viability at the highest concentration (10 µM) on HT-29 (58% (**3**) and 55% (**6**)) and MCF-7 cells (69% (**3**) and 53% (**6**)), demonstrating that all measured compounds displayed an in vitro cytotoxic activity. Nevertheless, the replacement of ^6^Asp by D-Asp, D-Glu or D-Trp led to a decreased cytostatic effect on the estimated cancer cell lines.

**Table 2:** In vitro cytostatic effects of GnRH-III bioconjugates on HT-29 human colon cancer and MCF-7 human breast cancer cell line.

code	[^8^Lys(Dau=Aoa)]–GnRH-III compound	MCF-7 IC_50_ [µM]	HT-29 IC_50_ [µM]

**K1**	[^6^L-Asp]	3.2 ± 0.1	1.5 ± 0.5
**1**	[^6^D-Asp]	13.0 ± 0.5	8.9 ± 1.3
**2**	[^6^D-Glu]	6.8 ± 1.0	10.1 ± 1.4
**3**	[^6^D-Trp]	n.d.^a^	n.d.^a^
**K2**	[^4^Lys(Bu),^6^L-Asp]	2.7 ± 0.1	1.9 ± 0.7
**4**	[^4^Lys(Bu),^6^D-Asp]	6.2 ± 0.2	9.3 ± 1.1
**5**	[^4^Lys(Bu),^6^D-Glu]	7.0 ± 1.2	13.7 ± 2.6
**6**	[^4^Lys(Bu),^6^D-Trp]	n.d.^a^	n.d.^a^

^a^n.d. - no data (compound **3** and **6** precipitated in medium at concentrations higher than 20 µM – no dose response). All values represent mean ± SE.

Considering the determined IC_50_ values, no relevant differences of the antiproliferative activity between the ^4^Ser and the corresponding ^4^Lys(Bu) derivatives could be observed which is not in line with our previous data [29]. This might be a result of the prolonged incubation time which was modified from 6 hours up to 24 hours because of the decreased antitumor activity of the novel compounds and the application of alamarBlue^®^ instead of MTT assay. It can be assumed that the ^4^Ser bioconjugates require a longer period of treatment to be fully effective. The D-Glu containing GnRH-III derivatives **2** and **4** had slightly higher IC_50_ values on HT-29 cells than the D-Asp compounds whereby the degradation profile of **2** and **4** displayed an enhanced release of the smallest Dau-containing metabolite indicating that the GnRH-III bioconjugates vary in their affinity for the GnRH-RI and/or their cellular uptake.

### Radioligand binding studies

To investigate the binding affinity of the novel compounds to the GnRH-receptor, an in vitro ligand competition assay has been performed on human pituitary and GnRH-R positive human prostate cancer tissues. Hereby, the displacement of radiolabeled triptorelin by the unlabeled bioconjugates **1**, **2**, **4** and **5** was determined. For a better comparison, the bioconjugates **K1** and **K2** were also analyzed and used as reference. The obtained results summarized in Table 3 point out that all bioconjugates replaced [^125^I]-triptorelin with IC_50_ values in low nanomolar range. With exception of conjugate **5** (7.9 nM and 11.6 nM), all evaluated compounds displayed slightly higher binding affinity on human prostate cancer (3.0–10.4 nM) than on human pituitary tissue (3.9–23.5 nM) being in accordance with our previous observations [29]. The lowest ligand concentration causing 50% inhibition of radioligand binding was obtained for **K2** with 3.9 nM on pituitary and 3.0 nM on prostate cancer tissues which is only slightly higher than the reported values for GnRH-I–[D-Lys^6^(Dau=Aoa)] (1.6 nM and 0.9 nM) and GnRH-II–[D-Lys^6^(Dau=Aoa)] (4.2 nM and 2.1 nM) [29,50].

Nevertheless, it has to be considered that the determined IC_50_ values are within a narrow, low nanomolar range and do not differ widely. Especially, the binding affinity on prostate cancer tissue deviate only slightly (3.0–11.6 nm) indicating that the incorporation of D-Asp or D-Glu in position 6 did not substantially affect the receptor binding properties of the GnRH-III derivatives. In addition, the selectivity of conjugates **1**, **2**, **4** is a bit higher toward the prostate cancer than the control conjugates **K1** and **K2**. According to our data, the incorporation of Lys(Bu) in position 4 instead of Ser increased the binding affinity but lowered the selectivity of the conjugates.

All investigated GnRH-III derivatives inhibited the binding of radiolabeled triptorelin efficiently by using increasing concentrations (1 pM to 1 µM). It has been reported that the GnRH-I derivatives cetrorelix and buserelin displaced [^125^I]-triptorelin completely by using the same concentration range, whereas GnRH-unrelated peptides (e.g., somatostatin-14, bombesin) could not inhibit the receptor binding at concentrations up to 1 µM [51–52]. Comparing our results with these findings from literature we can assume that the analyzed GnRH-III–Dau conjugates bind to the GnRH-receptor in a specific manner. However, the binding affinity data of the conjugates cannot explain alone the results of their in vitro antitumor activity.

**Table 3:** Competitive inhibition of [^125^I][^6^D-Trp]-GnRH-I binding to membranes of human pituitary and human prostate cancer specimens by GnRH-III–Dau-conjugates.

		IC_50_ [nM]	
		
code	[^8^Lys(Dau=Aoa)]–GnRH-III compound	pituitary	prostate cancer

**K1**	[^6^L-Asp]	6.3 ± 0.7	5.2 ± 0.6
**1**	[^6^D-Asp]	19.4 ± 2.8	8.9 ± 1.6
**2**	[^6^D-Glu]	23.5 ± 2.1	10.4 ± 1.3
**3**	[^6^D-Trp]	n.d.^a^	n.d.^a^
**K2**	[^4^Lys(Bu),^6^L-Asp]	3.9 ± 0.7	3.0 ± 1.1
**4**	[^4^Lys(Bu),^6^D-Asp]	6.1 ± 0.1	4.0 ± 1.3
**5**	[^4^Lys(Bu),^6^D-Glu]	7.9 ± 1.1	11.6 ± 2.0
**6**	[^4^Lys(Bu),^6^D-Trp]	n.d.^a^	n.d.^a^

^a^n.d. - no data. All values represent mean ± SE.

### Cellular uptake of the bioconjugates on MCF-7 human breast and HT-29 human colon cancer cells by flow cytometry

**Figure 4:**
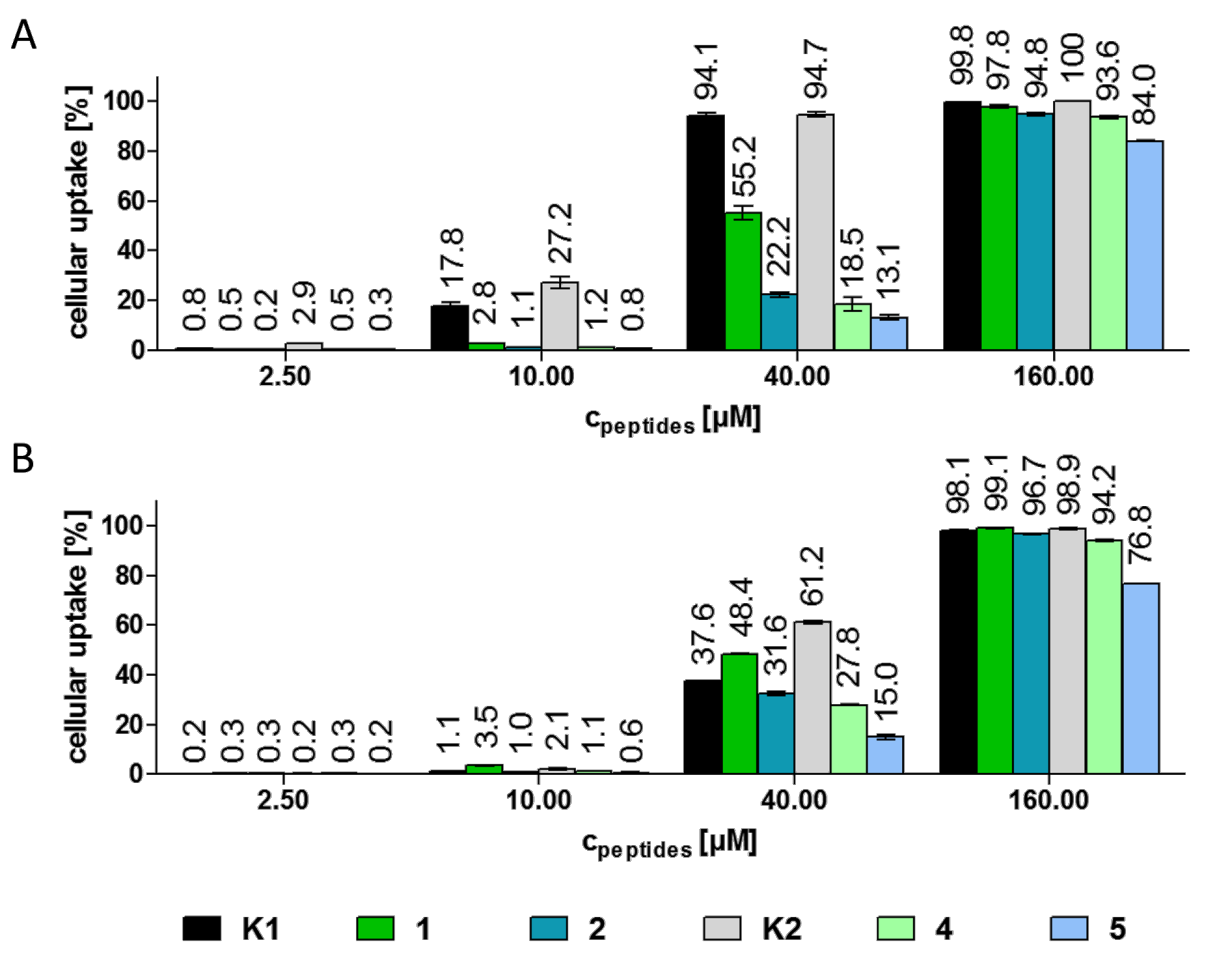
Cellular uptake of the GnRH-III bioconjugates at different concentrations on A) HT-29 and B) MCF-7 cells after 6 h determined by flow cytometry. Experiments were carried out in duplicates; error bars represent standard deviation.

The cellular uptake of the GnRH-III–drug conjugates was studied by flow cytometry on HT-29 and MCF-7 cancer cells (Figure 4). Due to their poor solubility in cell culture medium, the cellular uptake of the D-Trp containing compounds **3** and **6** was not investigated. The other conjugates were utilized in concentrations between 0.15–160 µM. For determination of the cellular uptake exclusively living cells were exploited. Because of the relatively low fluorescence intensity of Dau conjugates, on both cell lines a concentration of at least 2.5 µM was necessary to observe an increased uptake of the bioconjugates. In case of HT-29 cells, the two bioconjugates **K1** and **K2** which were used as internal standards displayed a higher cellular uptake than the new candidates. This became particularly obvious at lower concentrations (2.5 µM and 10 µM). In accordance with our previous data, the cellular uptake of **K2** by HT-29 cells is enhanced in comparison to **K1** [29,38]. Furthermore, the cellular uptake by HT-29 cells at 160 µM concentration was higher than 90% for all bioconjugates except compound **5** (83%). The same effect could be observed on MCF-7 cells, whereby the uptake of bioconjugate **5** was 76%. At 40 µM, **K2** was taken up by MCF-7 cells more effectively (61.2%) than the other compounds. Apart from that, bioconjugate **1** displayed a slightly higher uptake than **K1** at 10 µM and 40 µM concentration. In general, the bioconjugates with ^6^D-Asp had an improved cellular uptake in comparison with the corresponding ^6^D-Glu derivatives. Furthermore, in contrast to the control bioconjugates **K1** and **K2** the two compounds with Lys(Bu) in position 4 (**4** and **5**) revealed a declined cellular uptake over the ^4^Ser counterparts (**1** and **2**) on both cell lines. The differences in the behavior of the new compounds might be a result of the changed conformation of the D-amino acid containing derivatives. Due to the results of the receptor binding studies, we can assume that the binding site of the receptor is not essentially disturbed by the converted conformation, but it might be possible that the receptor internalization is influenced by the structure of the bound ligand.

Considering all these data, we can conclude that the cytostatic effect is not only influenced by the cellular uptake, but also the release of the effective metabolite plays an important role. For instance, the novel bioconjugate **1** provides the highest cellular uptake over the other D-amino acid containing derivatives, but the IC_50_ values are in the same range or even higher than the IC_50_ value of the other compounds **2**, **4** and **5**. It can be assumed that this effect occurred due to the reduced release of the smallest Dau-containing metabolite.

### Confocal laser scanning microscopy (CLSM) studies

**Figure 5:**
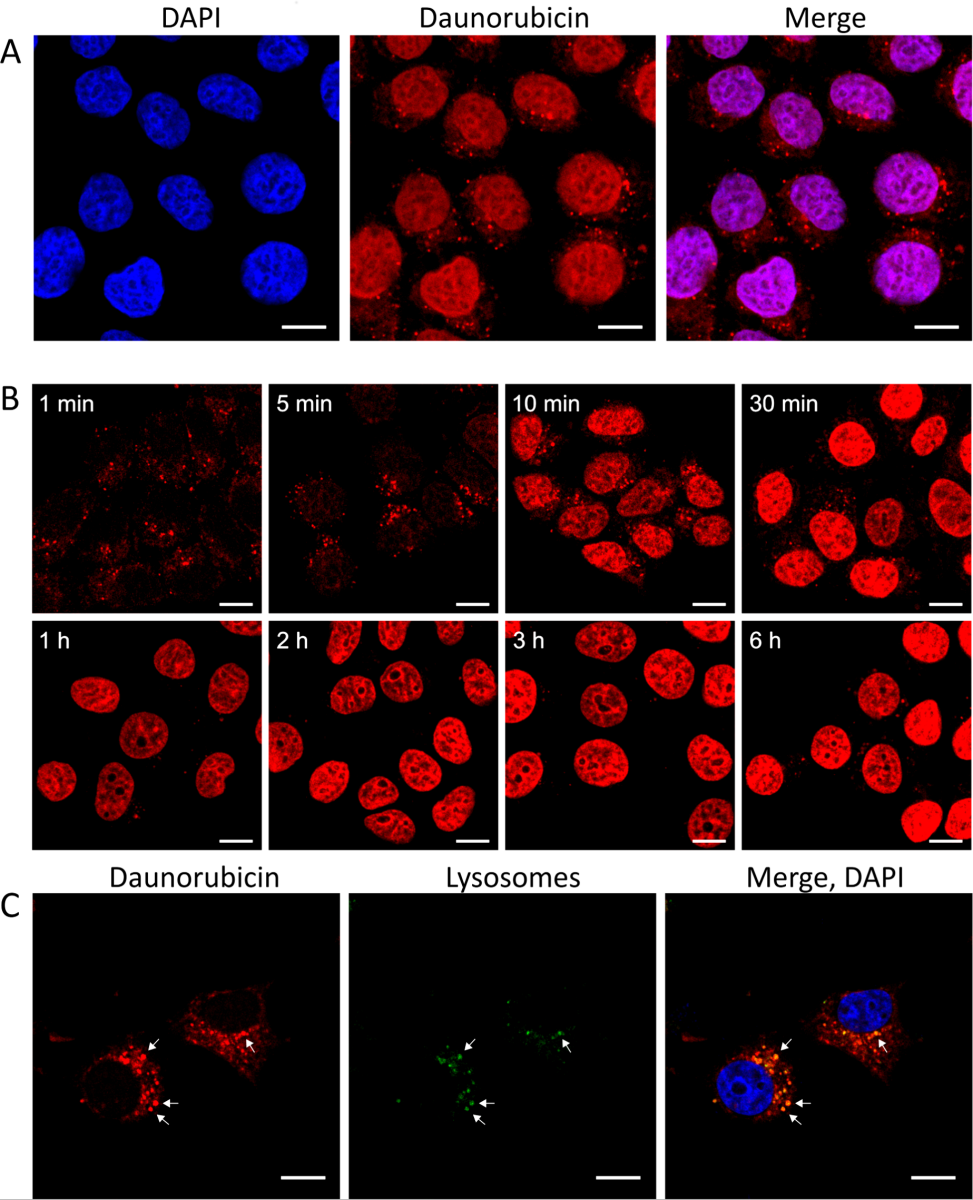
Cellular uptake of bioconjugate **K2** (40 µM) visualized by confocal laser scanning microscopy (CLSM) A) after 6 h incubation, daunorubicin (Dau) accumulates in the nucleus. B) Time dependent localization of bioconjugate **K2** after 1 min, 5 min, 10 min, 30 min, 1 h, 2 h, 3 h and 6 h incubation. C) Co-localization of **K2** (40 µM) with lysosomes (CytoPainter Lysosomal Staining Kit) after 5 min incubation. In the early stages of the cellular uptake the Dau signal is co-localized with the lysosomal staining (scale bars represent 10 µm).

Next to the quantitative analysis of the cellular uptake by flow cytometry, the cellular uptake and localization of **K1**, **K2**, **1**, **2**, **4** and **5** were studied on MCF-7 cells by CLSM. After 6 h incubation with the GnRH-III–Dau conjugates (*c* = 10 µM, 40 µM and 160 µM), MCF-7 cells were fixed and prepared for confocal laser scanning imaging. In order to gain insight into a possible co-localization with nuclei, DAPI-staining was performed. Images are displayed in BestFit mode to improve visualization of low signals and to optimize the image quality. The CLSM observations cannot be considered as quantitative analysis but provide qualitative information about subcellular localization. In case of all investigated compounds and concentrations, the Dau signal could be detected mainly in the nuclei and in small, cytosolic compartments (Figure 5A*,* Supporting Information File 1, Figures S9–S14) evidencing that the conjugated daunorubicin reaches its site of action.

To obtain more detailed information of the cellular mechanism after internalization, we analyzed the subcellular localization of one Dau conjugate at different timepoints by using shorter incubation times (Figure 5B). Due to the results achieved by flow cytometry and cell viability assay **K2** was selected for further investigations. The results indicate that Dau-containing metabolites can be found in high amount in the nuclei after already 10 to 30 minutes, whereas the CLSM images after 1 and 5 minutes display the Dau signal predominantly in small cytosolic vesicles.

We supposed that the smaller cytosolic compartments seen at early timepoints might be lysosomes. To prove this assumption, a lysosomal co-localization study was performed with **K2** on MCF-7 cells. After 5 min incubation, the detected Dau signals corresponded largely with the signal of the lysosomal stain revealing a co-localization with lysosomes (Figure 5C). The remaining vesicles which display only the Dau signal are assumed to be endosomes. Based on these findings it can be concluded that our lead compound **K2** is uptaken through an endocytic pathway.

### Receptor blockage by triptorelin

**Figure 6:**
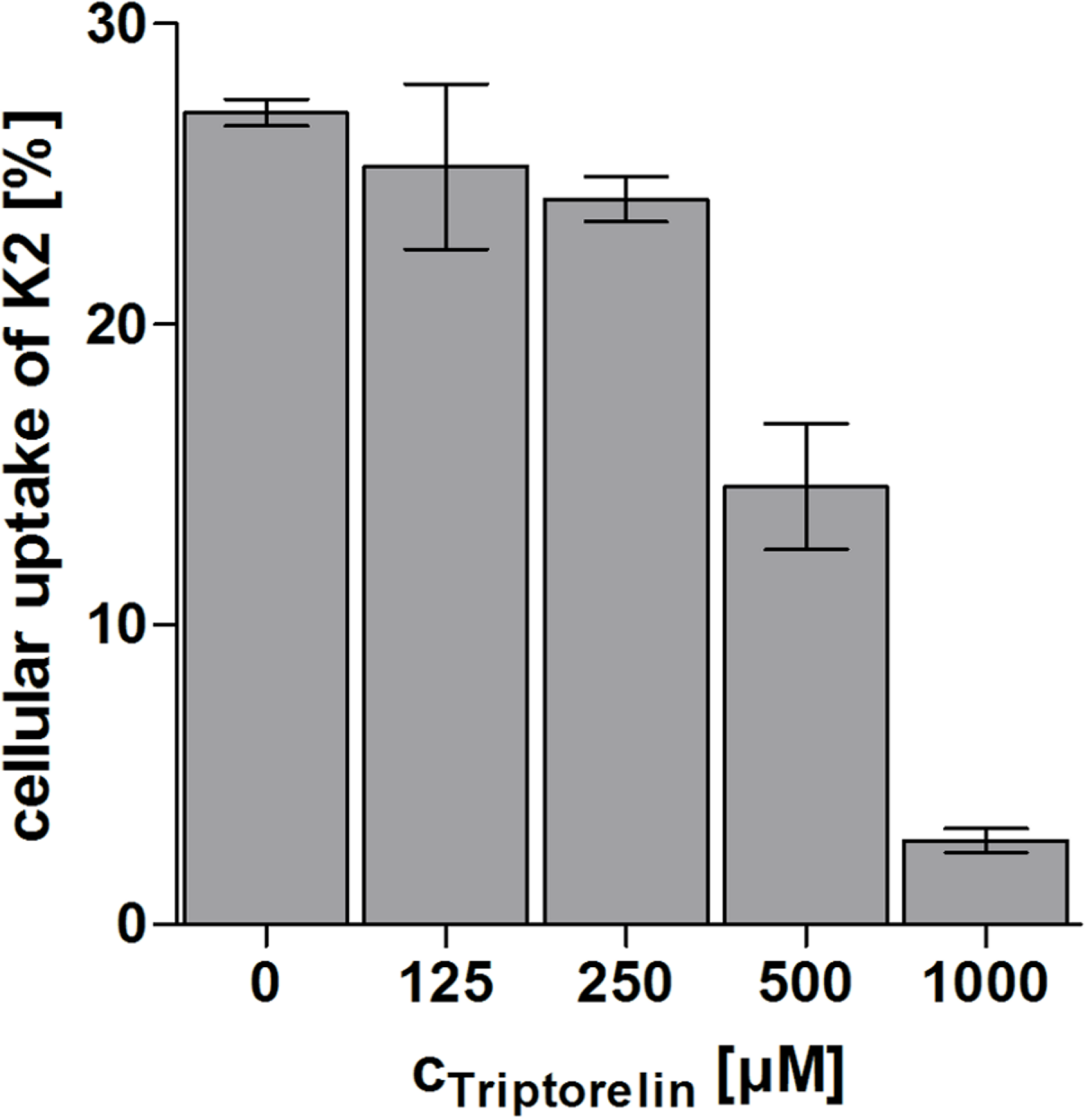
Competitive inhibition of the GnRH-R on MCF-7 cells. Cellular uptake of the GnRH-III bioconjugate **K2** (40 µM) was studied in the presence of superagonist triptorelin (125–1000 µM) by flow cytometry. Cells were treated simultaneously with **K2** and triptorelin for 100 min.

In order to investigate if the bioconjugate **K2** is uptaken in a receptor-mediated manner, a competition assay was performed on MCF-7 human breast cancer cells using an excess of the superagonist triptorelin. This GnRH-R binder was also used to prove the mechanism of action of the Dox–GnRH-I bioconjugate zoptarelin on Hec-1a and Ishikawa endometrial cancer cells [14]. To analyze the effect of triptorelin on the cellular uptake of the GnRH-III bioconjugates, MCF-7 cells were treated collectively with **K2** (fixed concentration of 40 µM) and triptorelin (concentration range of 125–1000 µM). Recently, Gründker et al. reported that triptorelin treatment leads to an increased density of GnRH-I receptors on MCF-7 cells [53]. Hence, our intention was to avoid long term incubation with triptorelin. The results of the CLSM studies indicate that 1–2 hours are sufficient to obtain substantial Dau uptake, therefore cells were treated for 100 min. Afterwards, the cells were analyzed by flow cytometry displaying a reduction of the cellular uptake of **K2** by an increasing triptorelin concentration (Figure 6). These results strongly indicate that the internalization of the Dau–GnRH-III bioconjugates can be blocked by triptorelin suggesting that the cellular uptake occurs in a GnRH-R-mediated manner.

## Conclusion

Our studies demonstrate that the cytostatic effect of drug bioconjugates, in our case Dau–GnRH-III derivative conjugates, depend on various cellular events. Thus, the efficiency of DDSs is not only defined by their stability under physiological conditions and the selectivity to cancer cells, but also the cellular uptake of the drug–carrier molecule and the release of the active agent play an important role.

All investigated compounds exhibit cytostatic effects on HT-29 human colon cancer and MCF-7 human breast cancer cells, whereby the well-defined lead compound **K2** remains our most promising drug candidate. The six novel GnRH-III-[^6^D-Aaa]–Dau conjugates reveal 3–5 times lower antitumor activity than the parent conjugates. Their antitumor effects are influenced by many factors which counteract each other. Therefore, no significant difference in IC_50_ values could be observed. It turned out that the ^4^Lys(Bu)-containing conjugates **4** and **5** have a higher binding affinity to the GnRH receptors in comparison to the ^4^Ser-containing ones. Furthermore, the D-Asp derivatives **1** and **4** show slightly improved receptor binding properties than the D-Glu derivatives **2** and **5** which might be the reason for the enhanced cellular uptake of the D-Asp conjugates. However, our lysosomal degradation studies pointed out that the digestion of conjugates **1** and **4** is less effective and results in larger metabolites. These metabolites might have a reduced DNA intercalating potency than the smallest Dau-metabolite (H-Lys(Dau=Aoa)-OH) which could be identified in case of the control peptides **K1** and **K2**. It is worth mentioning that in case of D-Trp containing conjugates a significant amount of H-Lys(Dau=Aoa)-OH metabolite was released, however, other biological study could not be made with them because of their poor solubility in cell culture medium. Due to this observation, the conjugates **3** and **6** might be interesting for further development. Nevertheless, our results provide significant information about the influence of the cellular uptake and the release of the effective metabolite on the efficiency of the DDS.

Next to these findings, we could systematically specify the receptor-mediated endocytosis pathway for GnRH-III-[^4^Lys(Bu), ^8^Lys(Dau=Aoa)] which is also representative for other Dau–GnRH-III bioconjugates. Based on our results, we can assume that the GnRH-III bioconjugates specifically bind the GnRH-Rs on cancer cells and induce in that way their internalization. Moreover, the outcome of the time dependent localization of Dau peptide conjugates or the Dau-containing metabolites can give clear indication for an endocytotic pathway. Furthermore, we could evident the localization of Dau–GnRH-III conjugates in lysosomes by lysosomal staining. All CLSM data attest the intranuclear accumulation of Dau proving the presence of Dau-containing metabolites on its site of action.

In summary, our findings support the development of new therapeutic approaches based on new cytotoxic peptide conjugates targeting GnRH receptors in human cancers.

## Experimental

### Material

All amino acid derivatives and Rink-Amide MBHA resin were purchased from Iris Biotech GmBH (Marktredwitz, Germany). Boc-aminooxyacetic acid (Boc-Aoa-OH), aminooxyacetic acid, scavengers, coupling agents and cleavage reagents (1-hydroxybenzotriazole hydrate (HOBt), *N,N’*-diisopropylcarbodiimide (DIC), triisopropylsilane (TIS), piperidine, 1,8-diazabicyclo[5.4.0]undec-7-ene (DBU), trifluoroacetic acid (TFA), diisopropylethylamine (DIPEA), acetic anhydride (Ac_2_O), methanol (MeOH), *n*-butyric anhydride and solvent for HPLC (acetonitrile (ACN)) were obtained from Sigma-Aldrich Kft (Budapest, Hungary). Daunorubicin hydrochloride was a gift from IVAX (Budapest, Hungary). *N,N*-Dimethylformamide (DMF), dichloromethane (DCM) and diethyl ether (Et_2_O) were purchased from Molar Chemicals Kft (Budapest, Hungary). All reagents and solvents were of analytical grade or highest available purity.

### Synthesis of oxime bond-linked GnRH-III–[^4^Ser/^4^Lys(Bu), ^6^Aaa, ^8^Lys(Dau=Aoa)] bioconjugates

The Dau–GnRH-III derivatives were synthesized by solid phase peptide synthesis according to Fmoc/*t*-Bu chemistry on a Rink-Amide MBHA resin (0.73 mmol/g coupling capacity) followed by ligation of Dau (oxime bond) in solution. All peptides were synthesized manually by usage of the following Fmoc-protected amino acid derivatives: Fmoc-Gly-OH, Fmoc-Pro-OH, Fmoc-Lys(Mtt)-OH, Fmoc-Lys(Dde)-OH, Fmoc-Trp-OH, Fmoc-D-Trp-OH, Fmoc-Asp(O*t-*Bu)-OH, Fmoc-D-Asp(O*t*-Bu)-OH, Fmoc-D-Glu(O*t*-Bu)-OH, Fmoc-His(Trt)-OH and Fmoc-Ser(*t*-Bu)-OH. Pyroglutamic acid (Glp or <E) was attached to the peptide chain without any protection. The general protocol for the synthesis started with DMF-washing (4 × 1 min), followed by Fmoc deprotection with 2% piperidine, 2% DBU in DMF (4 times; 2 + 2 + 5 + 10 min). The coupling reaction was performed by using 3 equiv of α-Fmoc-protected amino acid derivative, 3 equiv DIC and 3 equiv HOBt in DMF (60 min). After washing with DMF (3 × 1 min) and DCM (2 × 1 min) the success of the coupling was controlled by ninhydrine test. After assembly of the protected decapeptide the Dde group of ^4^Lys was removed with 2% hydrazine in DMF (12 × 5 min) and the peptidyl-resin was washed with DMF (5 × 1 min). Afterwards, the ε-NH_2_ amino group was butyrylated with 3 equiv butyric anhydride and 3 equiv DIPEA in DMF (2 h). In the next step, ^8^Lys(Mtt) was deprotected with 2% TFA in DCM (6 × 5 min). Then the resin-bound peptide was neutralized with 10% DIPEA in DCM (3 × 5 min) and Boc-Aoa-OH was coupled for 2 h using DIC, HOBt coupling reagents (3 equiv each to the amino group). The treatment with 95% TFA, 2.5% TIS and 2.5% water (v/v/v) in the presence of 10 equiv free aminooxyacetic acid as “carbonyl capture” reagent (2 h, at room temperature (rt)) resulted in the simultaneous removal of the side chain protecting groups and the cleavage of the peptide from the resin [54]. Peptides were isolated by precipitation with ice-cold Et_2_O, centrifuged, washed 3 times, dissolved in water/ACN (0.1% TFA) 4:1 (v/v) and lyophilized. Subsequent to the purification of the crude peptides by RP-HPLC, the solvent was evaporated and Dau was conjugated to the aminooxyacetylated ^8^Lys. The oxime bond formation was carried out in 0.2 M ammonium acetate buffer (pH 5.0), at a peptide concentration of 10 mg/mL and 1.3 equiv Dau [21]. The reaction mixtures were stirred overnight at rt and then purified by RP-HPLC. The resulting GnRH-III bioconjugates were characterized by analytical RP-HPLC and MS.

### RP-HPLC

The crude peptides and the bioconjugates were purified on a KNAUER 2501 HPLC system (H. Knauer, Bad Homburg, Germany) using a preparative Phenomenex Luna C18(2) column (100 Å, 10 µm, 250 mm × 21.2 mm) (Torrance, CA, USA). Linear gradient elution (0 min 20% B; 5 min 20% B; 50 min 80% B) with eluent A (0.1% TFA in water) and eluent B (0.1% TFA in ACN/H_2_O (80:20, v/v)) was used at a flow rate of 9.5 mL/min. Peaks were detected at 280 nm.

Analytical RP-HPLC was performed on a KNAUER 2501 HPLC system using a Phenomenex Luna C18 column (100 Å, 5 µm, 250 mm × 4.6 mm) as a stationary phase. Linear gradient elution (0 min 0% B; 5 min 0% B; 50 min 90% B) at a flow rate of 1 mL/min with eluents described above. Peaks were detected at 220 nm.

### Mass spectrometry

Electrospray ionization (ESI) mass spectrometric analyses were carried out on an Esquire 3000+ ion trap mass spectrometer (Bruker Daltonics, Bremen, Germany). Spectra were acquired in the 50–2500 *m*/*z* range. Samples were dissolved in a mixture of ACN/water (1:1, v/v) and 0.1% formic acid.

Liquid chromatography–mass spectrometry (LC–MS) was carried out on the same spectrometer equipped with an Agilent 1100 HPLC system and a diode array detector (Agilent, Waldbronn, Germany). Peptides were separated on a Supelco C18 column (150 mm × 2.1 mm, 3 µm) (Hesperia, CA) using a linear gradient from 2–70% B in 25 min (eluent A: ddH_2_O, 0.1% HCOOH; eluent B: 80% ACN, 0.1% HCOOH at a flow rate of 0.2 mL/min). Spectra were recorded in positive ion mode in the 100–2500 *m*/*z* range.

### Electronic circular dichroism spectroscopy

Electronic circular dichroism (ECD) spectra were recorded on a Jasco J-810 (Jasco International Co., Ltd., Tokyo, Japan) spectropolarimeter at 25 °C in quartz cells of 0.02 cm path length, under constant nitrogen flush. The instrument was calibrated with 0.06% (w/v) ammonium-D-camphor-10-sulfonate (Katayama Chemical, Japan) in water. The bioconjugates were dissolved in water (*c* = 340 µM). The spectra show averages of six scans in a wavelength range of 185–260 nm. The results were expressed in terms of mean molar ellipticity (deg cm^2^ dmol^−1^) after subtracting the solvent baseline.

### Degradation of GnRH-III bioconjugates in rat liver lysosomal homogenate

The rat liver lysosomal homogenate was prepared as previously described [26]. The protein concentration was determined by Qubit Protein Assay Kit according to the manufacturer’s protocol (ThermoFisher Scientific, Waltham, MA, USA). The bioconjugates were dissolved in ddH_2_O to a concentration of 5 µg/µL. The reaction was carried out in 0.2 M NaOAc buffer (pH 5), with an identical concentration of bioconjugate and rat liver lysosomal homogenate (0.25 µg/µL). The reaction mixtures were incubated at 37 °C and aliquots of 15 µL were taken at 5 min, 1 h, 2 h, 4 h, 8 h and 24 h and quenched with 2 µL of acetic acid. The analysis of the samples was performed by LC–MS.

### Cell culture

MCF-7 human breast adenocarcinoma cells were cultured in Dulbecco’s Modified Eagle Medium (DMEM, Lonza, Basel, Switzerland), supplemented with 10% (v/v) fetal bovine serum (FBS, Lonza), L-glutamine (2 mM, Lonza), non-essential amino acids (NEAA, Sigma-Aldrich Kft), sodium pyruvate (1 mM, Lonza) and penicillin-streptomycine (Lonza). HT-29 human colon adenocarcinoma cells were cultured in RPMI-1640 (Lonza), supplemented with FBS, L-glutamine and penicillin-streptomycine. Cells were maintained in plastic culture dishes at 37 °C with a humidified atmosphere containing 5% CO_2_/95% air.

### Stability of GnRH-III bioconjugates in cell culture medium

The GnRH-III bioconjugates were dissolved in water to a concentration of 2.5 mg/mL followed by the dilution with serum-free cell culture medium (final bioconjugate concentration: 0.5 mg/mL). The mixtures were incubated at 37 °C for 24 h and samples of 50 µL were directly monitored by RP-HPLC at time points 0 h, 1 h, 2 h, 6 h and 24 h.

### In vitro cytostatic effect studies

Cells were seeded to 96-well plates (Sarstedt, Nümbrecht, Germany, 5 × 10^3^ cells/well) one day prior to the treatment in complete cell medium. Cells were treated with bioconjugates in serum-free medium (concentration range 0.07−150 µM, control wells were treated with serum-free medium). After 24 h, cells were washed two times with serum-free medium and were incubated in complete medium for 48 h. Cytostasis detection was performed using alamarBlue reagent^®^ (ThermoFisher Scientific) according to the manufacturer’s instructions. Fluorescence was detected using Synergy H4 multi-mode microplate reader (BioTek, Winooski, VT, USA), excitation and emission wavelengths were set to 570 and 620 nm, respectively. Cytostasis was measured using 4 parallels, each experiment was repeated twice. Cytostatic effect (and IC_50_ values) were calculated by using nonlinear regression (sigmoidal dose response) with Origin Pro8 (OriginLab Corp., Northampton, MA, USA.).

### Cellular uptake determination by flow cytometry

For determining cellular uptake of the bioconjugates, cells were seeded to 24-well plates (Sarstedt) one day prior to the experiment (10^5^ cells/well) in complete cell medium. Treatment with Dau-conjugated peptides was performed in serum-free culture medium for 6 h, concentrations ranging from 0.15–160 µM. In case of the competitive inhibition of the GnRH-Rs, MCF-7 cells were simultaneously treated with triptorelin and **K2** for 100 min, whereby triptorelin was used in a concentration range of 125–1000 µM and **K2** was applied in a fixed concentration of 40 µM. After treatment, cells were washed two times with serum-free medium and one time with HPMI medium (containing 100 mM NaCl, 5.4 mM KCl, 0.4 mM MgCl_2_, 0.04 mM CaCl_2_, 10 mM Hepes, 20 mM glucose, 24 mM NaHCO_3_ and 5 mM Na_2_HPO_4_ at pH 7.4) and trypsinized for 10 min at 37 °C. Trypsinization was stopped by HPMI medium supplemented with 10% FBS. Detached cells were centrifuged at 216*g* for 5 min at 4 °C and the supernatant was removed. Cells were resuspended in HPMI medium, intracellular fluorescence intensity (that is proportional to the cellular uptake) was monitored by flow cytometer BD LSR II (BD Bioscience, Franklin Lakes, NJ, USA). Data were analyzed by FACSDiVa (BD Bioscience) 5.0 software.

### Confocal microscopy imaging

Cells were seeded to cover glass containing (thickness 1, Assistant, Karl Hecht GmbH & Co KG, Sondheim/Rhön Germany) 24-well plates one day prior to the experiment in complete cell medium. Treatment was performed in serum-free medium for the indicated incubation time. Cells were washed two times with phosphate buffered saline (PBS) and fixed by 4% paraformaldehyde for 20 min at 37 °C. After three times washing with PBS, nuclei were stained by 4′,6-diamidine-2-phenylindole dihydrochloride (DAPI, 0.2 µg/mL, dissolved in PBS, Sigma-Aldrich Kft.) for 15 min. After washing, cover glasses were mounted to microscopy slides (VWR International, Debrecen, Hungary) by Mowiol^®^ 4-88 mounting medium (Sigma-Aldrich Kft.). In case of the lysosomal co-localisation study, lysosomes were stained in living cells before treatment with peptide **K2** by CytoPainter Lysosomal Staining Kit - Deep Red Fluorescence (abcam, Cambridge, UK), according to the manufacturer’s instructions. Confocal microscopy was carried out using a Zeiss LSM 710 system (Carl Zeiss Microscopy GmbH, Jena, Germany) with a 40× oil objective. Images were processed by software ZEN Lite (Carl Zeiss Microscopy GmbH).

### Radioligand binding studies

Radiolabeled triptorelin was used for displacement studies to evaluate the binding affinity of the conjugates **K1**, **K2**, **1**, **2**, **4** and **5** to GnRH-RI on human pituitary and human prostate cancer cells. Tissue samples derived by autopsy from normal human pituitary (anterior lobe) and human prostate cancer cells were obtained from a patient at the time of initial surgical treatment. The collection and the use of these specimens for our studies are approved by the local Institutional Ethics Committee. Membranes for receptor binding studies have been prepared as previously described [29,51–52,55]. Radioiodinated GnRH-I agonist triptorelin was prepared by chloramines-T method and purified by RP-HPLC [29,51–52,56]. This radioligand has been well-characterized and shows high-affinity binding to human and rat pituitaries as well as human breast, prostate, and other cancers [51–52]. The binding affinities of the nonradio-labeled GnRH-III bioconjugates to GnRH-RI were determined by displacement of [^125^I]-GnRH-I-[^6^D-Trp] performing an in vitro ligand competition assay as recently reported [29,51–52]. Hereby, membrane homogenates which contain 50–160 mg protein were incubated in duplicate or triplicate with 60–80,000 cpm [^125^I]-GnRH-I-[^6^D-Trp] and increasing concentration (1 pM–1 µM) of nonradioactive bioconjugates as competitors in a total volume of 150 mL of binding buffer. After incubation, 125 mL aliquots of suspension were transferred onto the top of 1 mL of ice-cold binding buffer which contained 1.5% bovine serum albumin in siliconized polypropylene microcentrifuge tubes (Sigma-Aldrich Kft.). The tubes were centrifuged at 12,000*g* for 3 min at 4 °C (Beckman J2-21M, Beckman Coulter, Inc., Brea, CA). Supernatants were aspirated and the bottoms of the tubes containing the pellet were cut off and counted in a gamma counter. Protein concentration was determined by the method of Bradford using a Bio-Rad protein assay kit (Bio-Rad Laboratories, USA). The LIGAND-PC computerized curve-fitting program of Munson and Rodbard was used to determine the receptor binding characteristics and IC_50_ values [29,51–52].

## Supporting Information

File 1 Characterization data for compounds **1–6**: RP-HPLC chromatograms and ESI–MS spectra; fragments of **1–6** produced by lysosomal rat liver homogenate; cellular uptake of **K1**, **K2**, **1**, **2**, **4**, **5** by CLSM.
