# Supplementary material for: Synthesis and in vitro biochemical evaluation of oxime bond-linked daunorubicin–GnRH-III conjugates developed for targeted drug delivery
Source: Beilstein J Org Chem. 2018 Apr 4;14:756–71. doi: 10.3762/bjoc.14.64 (PMC5905287; doi:10.3762/bjoc.14.64)
Supplement: File 1 — Characterization data for compounds 1–6: RP-HPLC chromatograms and ESI–MS spectra; fragments of 1–6 produced by lysosomal rat liver homogenate; cellular uptake of K1, K2, 1, 2, 4, 5 by CLSM. [file Beilstein_J_Org_Chem-14-756-s001.pdf]

Supporting Information

for

Synthesis and in vitro biochemical evaluation of oxime bond linked daunorubicin–GnRH-III conjugates developed for targeted drug delivery

Sabine Schuster[1,2], Beáta Biri-Kovács[1,2], Bálint Szeder[3], Viktor Farkas[4], László Buday[3], Zsuzsanna Szabó[5], Gábor Halmos[5] and Gábor Mező[1,2]*

Address: [1]MTA-ELTE Research Group of Peptide Chemistry, Hungarian Academy of Sciences, Eötvös L. University, 1117 Budapest, Hungary, [2]Institute of Chemistry, Eötvös L. University, 1117 Budapest, Hungary, [3]Research Centre for Natural Sciences, Institute of Enzymology, Hungarian Academy of Sciences, 1117 Budapest, Hungary, [4]MTA-ELTE Protein Modelling Research Group, Hungarian Academy of Sciences, Eötvös L. University, 1117 Budapest, Hungary and [5]Department of Biopharmacy, Faculty of Pharmacy, University of Debrecen, 4032 Debrecen, Hungary

Email: Gábor Mező - gmezo@elte.hu

*Corresponding author

Characterization data for compounds 1–6: RP-HPLC chromatograms and ESI–MS spectra; fragments of 1–6 produced by lysosomal rat liver homogenate; cellular uptake of K1, K2, 1, 2, 4, 5 by CLSM

Table of contents

RP-HPLC profile and ESI-ion trap mass spectrum of GnRH-III-[^{4}Ser, ^{8}Lys(Dau=Aoa)] (**K1**) S3

RP-HPLC profile and ESI-ion trap mass spectrum of GnRH-III-[^{4}Lys(Bu), ^{8}Lys(Dau=Aoa)] (**K2**) S3

RP-HPLC profile and ESI-ion trap mass spectrum of GnRH-III-[^{4}Ser, ^{6}D-Asp, ^{8}Lys(Dau=Aoa)] (**1**) S4

RP-HPLC profile and ESI-ion trap mass spectrum of GnRH-III-[^{4}Ser, ^{6}D-Glu, ^{8}Lys(Dau=Aoa)] (**2**) S4

RP-HPLC profile and ESI-ion trap mass spectrum of GnRH-III-[^{4}Ser, ^{6}D-Trp, ^{8}Lys(Dau=Aoa)] (**3**) S4

RP-HPLC profile and ESI-ion trap mass spectrum of GnRH-III-[^{4}Lys(Bu), ^{6}D-Asp, ^{8}Lys(Dau=Aoa)] (**4**) S5

RP-HPLC profile and ESI-ion trap mass spectrum of GnRH-III-[^{4}Lys(Bu), ^{6}D-Glu, ^{8}Lys(Dau=Aoa)] (**5**) S5

RP-HPLC profile and ESI-ion trap mass spectrum of GnRH-III-[^{4}Lys(Bu), ^{6}D-Trp, ^{8}Lys(Dau=Aoa)] (**6**) S5

Fragments produced by the cleavage of GnRH-III-[^{4}Lys(Bu)/^{4}Ser, ^{6}Aaa, ^{8}Lys(Dau=Aoa)] bioconjugates in the presence of rat liver homogenate S6

Cellular uptake of **K1** by confocal laser scanning microscopy S8

Cellular uptake of **1** by confocal laser scanning microscopy S9

Cellular uptake of **2** by confocal laser scanning microscopy S10

Cellular uptake of **K2** by confocal laser scanning microscopy S11

Cellular uptake of **4** by confocal laser scanning microscopy S12

Cellular uptake of **5** by confocal laser scanning microscopy S13

Electronic circular dichroism spectra of bioconjugates S14

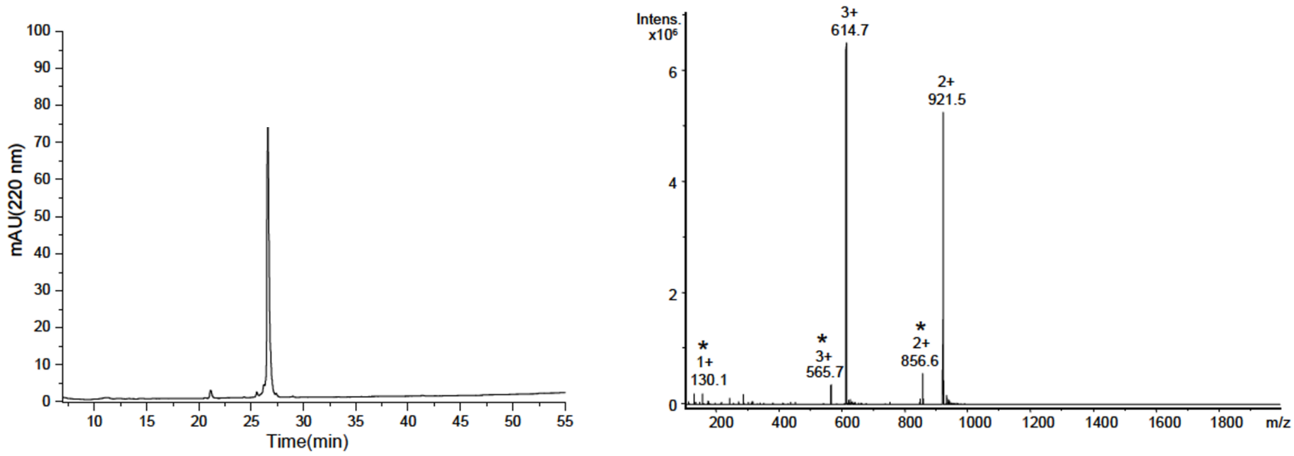

Figure S1. RP-HPLC profile and ESI-ion trap mass spectrum of GnRH-III–[[4]Ser, [8]Lys(Dau=Aoa)] (**K1**). (MW_{cal} /MW_{exp} = 1841.89/1841.05 g/mol, *fragment ion: amino sugar loss of Dau)

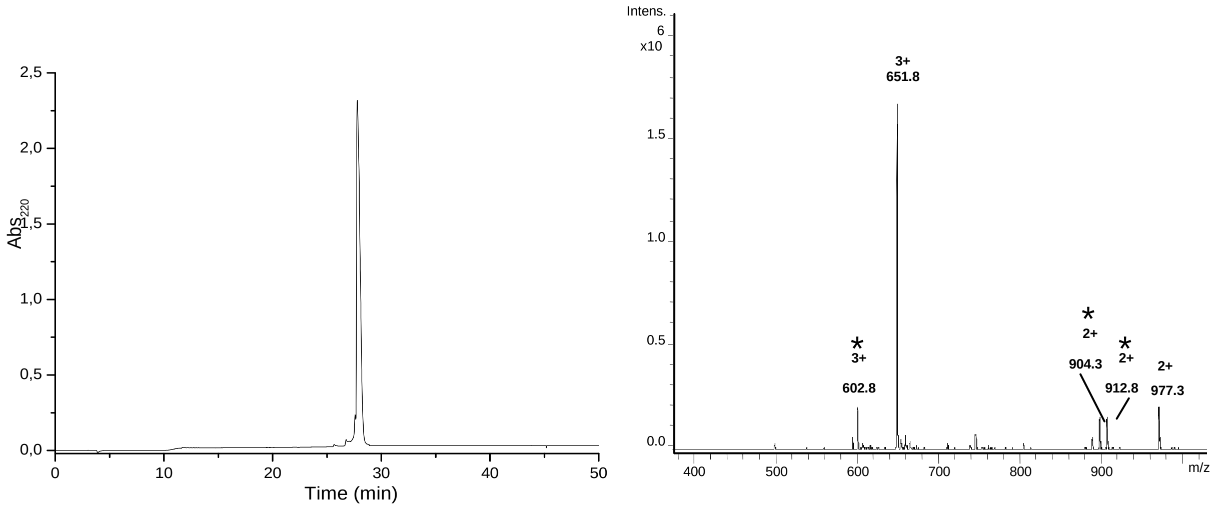

Figure S2. RP-HPLC profile and ESI-ion trap mass spectrum of GnRH-III–[[4]Lys(Bu), [8]Lys(Dau=Aoa)] (**K2**). (MW_{cal} /MW_{exp} = 1953.07/1952.70 g/mol).

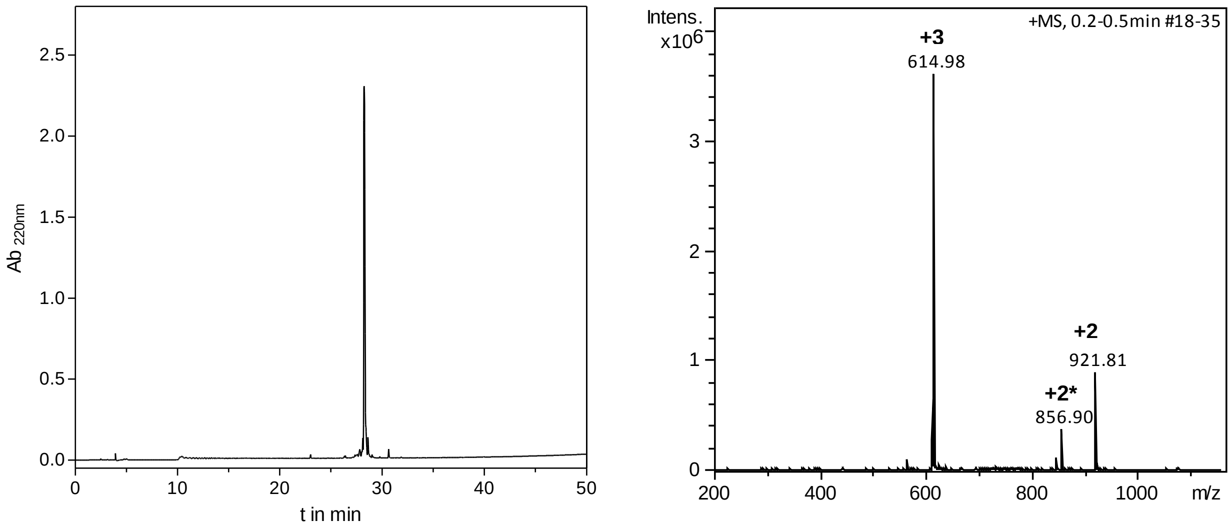

Figure S3. RP-HPLC profile and ESI-ion trap mass spectrum of GnRH-III–[[4]Ser, [6]D-Asp, [8]Lys(Dau=Aoa)] (**1**) (MW_{cal} /MW_{exp} = 1841.89/1841.60 g/mol, *fragment ion: amino sugar loss of Dau)

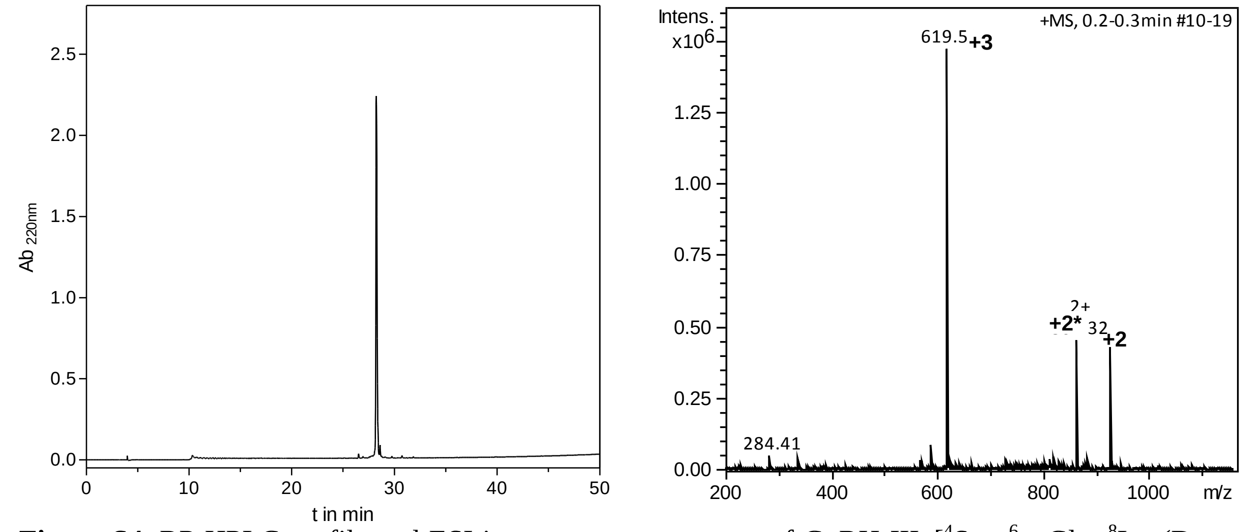

Figure S4. RP-HPLC profile and ESI-ion trap mass spectrum of GnRH-III–[[4]Ser, [6]D-Glu, [8]Lys(Dau=Aoa)] (**2**) (MW_{cal} /MW_{exp} = 1855.91/1855.70 g/mol)

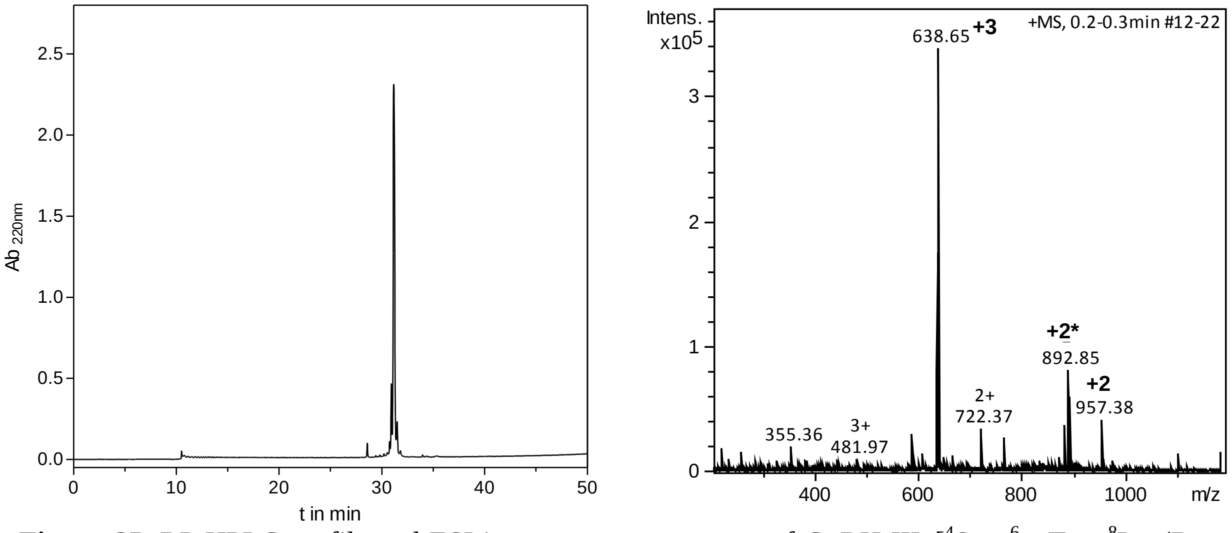

Figure S5. RP-HPLC profile and ESI-ion trap mass spectrum of GnRH-III–[[4]Ser, [6]D-Trp, [8]Lys(Dau=Aoa)] (**3**) (MW_{cal} /MW_{exp} = 1913.01/1912.80 g/mol)

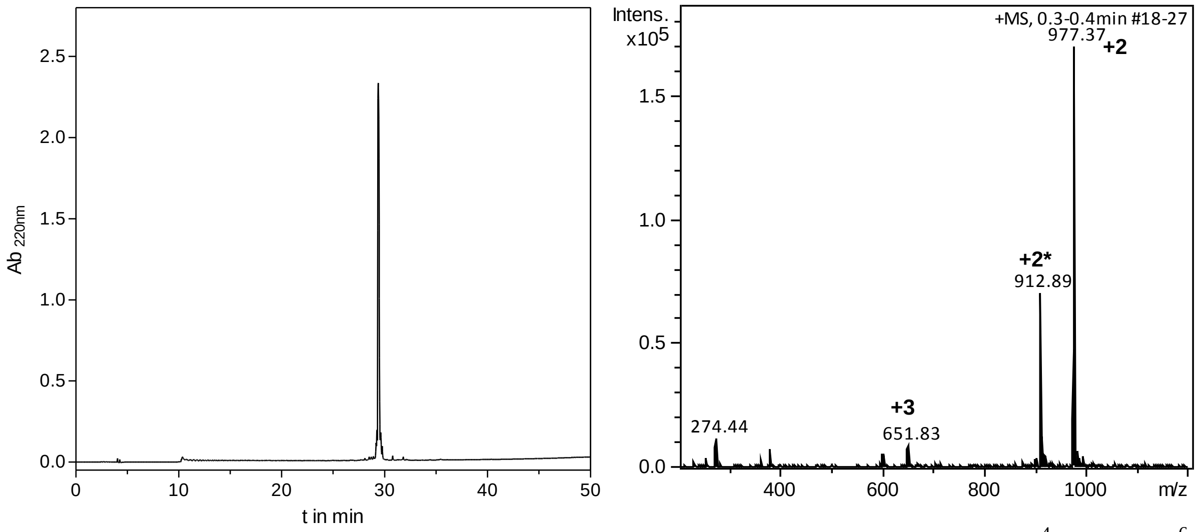

Figure S6. RP-HPLC profile and ESI-ion trap mass spectrum of GnRH-III–[[4]Lys(Bu), [6]D-Asp, [8]Lys(Dau=Aoa)] (**4**) (MW_{cal} /MW_{exp} = 1953.07/1952.90 g/mol)

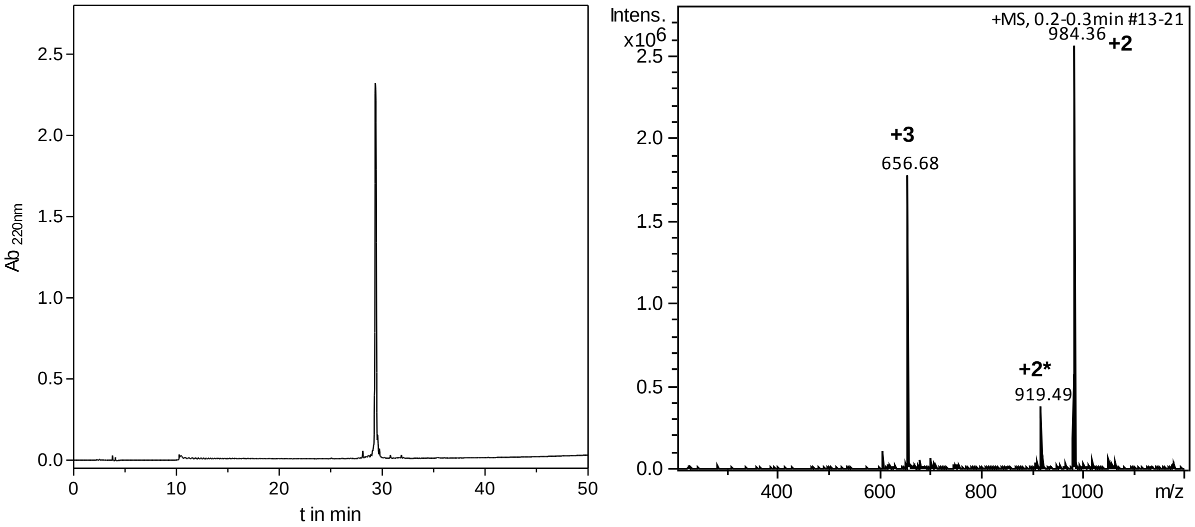

Figure S7. RP-HPLC profile and ESI-ion trap mass spectrum of GnRH-III–[[4]Lys(Bu), [6]D-Glu, [8]Lys(Dau=Aoa)] (**5**) (MW_{cal} /MW_{exp} = 1966.93/1966.70 g/mol)

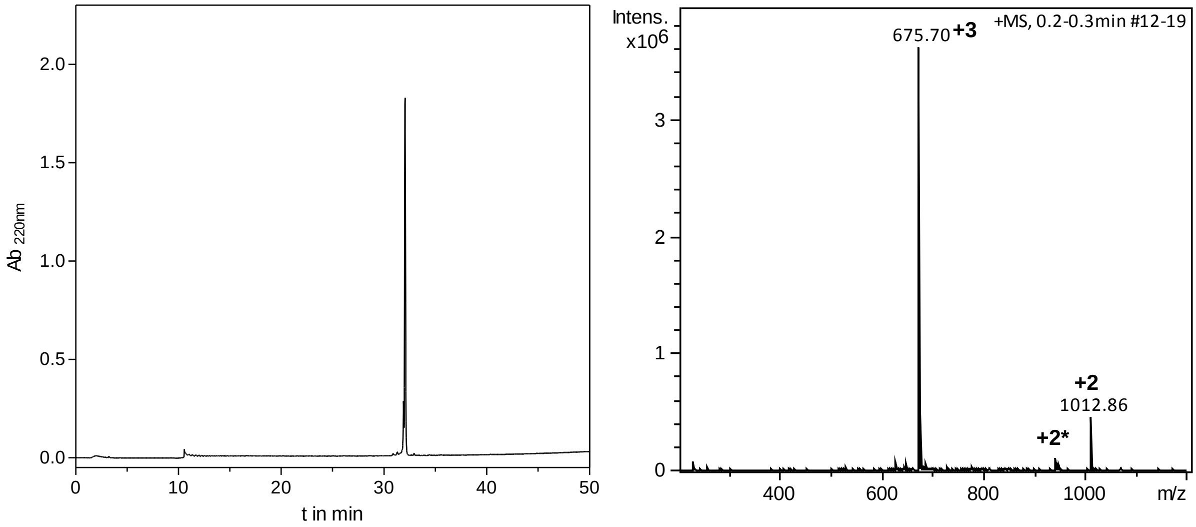

Figure S8. RP-HPLC profile and ESI-ion trap mass spectrum of GnRH-III–[[4]Lys(Bu), [6]D-Trp, [8]Lys(Dau=Aoa)] (**6**) (MW_{cal} /MW_{exp} = 2024.03/2023.70)

Table S1. Fragments produced by the cleavage of GnRH-III-[^{4}Lys(Bu)/^{4}Ser, ^{6}Aaa, ^{8}Lys(Dau=Aoa)] bioconjugates in the presence of rat liver homogenate

Code	Compound	Fragment	MW$_{cal}$/MW$_{exp}$
**K1**	[^{8}Lys(Dau=Aoa)]	<EHWSHDWK(Dau=Aoa)PG-NH$_2$	1841.89/1841.66
		<EHWSHDWK(Dau=Aoa)P-OH	1785.82/1785.27
		<EHWSHDWK(Dau=Aoa)-OH	1688.70/1688.69
		H-WSHDWK(Dau=Aoa)-OH	1440.49/1439.78
		H-HDWK(Dau=Aoa)PG-NH$_2$	1320.36/139.83
		H-HDWK(Dau=Aoa)-OH	1167.18/1166.87
		H-K(Dau=Aoa)PG-NH$_2$	881.94/881.39
		H-K(Dau=Aoa)P-OH	825.86/825.35
		H-K(Dau=Aoa)-OH	728.75/728.34
		<EHWSH-OH	676.68/676.23
		<EHWS-OH	539.54/539.32
		<EHW-OH	452.46/452.32
		H-DW-OH	319.12/319.28
**1**	[^{6}D-Asp, ^{8}Lys(Dau=Aoa)]	<EHWSHdWK(Dau=Aoa)PG-NH$_2$	1841.89/1841.63
		<EHWSHdWK(Dau=Aoa)P-OH	1785.82/1785.63
		<EHWSHdWK(Dau=Aoa)-OH	1688.70/1688.44
		H-WSHdWK(Dau=Aoa)PG-NH$_2$	1593.65/1593.61
		H-WSHdWK(Dau=Aoa)-OH	1440.49/1440.45
		H-SHdWK(Dau=Aoa)-OH	1254.26/1253.83
**2**	[^{6}D-Glu, ^{8}Lys(Dau=Aoa)]	<EHWSHeWK(Dau=Aoa)PG-NH$_2$	1855.90/1855.67
		<EHWSHeWK(Dau=Aoa)P-OH	1799.87/1799.79
		<EHWSHeWK(Dau=Aoa)-OH	1702.76/1701.85
		H-WSHeWK(Dau=Aoa)PG-NH$_2$	1607.70/1607.63
		H-WSHeWK(Dau=Aoa)-OH	1454.51/1453.91
		H-SHeWK(Dau=Aoa)-OH	1268.30/1267.75
		H-K(Dau=Aoa)-OH	728.75/728.33
**3**	[^{6}D-Trp, ^{8}Lys(Dau=Aoa)]	<EHWSHwWK(Dau=Aoa)PG-NH$_2$	1913.04/1912.79
		<EHWSHwWK(Dau=Aoa)P-OH	1856.941856.64
		<EHWSHwWK(Dau=Aoa)-OH	1759.85/1759.61
		H-WSHwWK(Dau=Aoa)-OH	1511.61/1511.58
		H-SHwWK(Dau=Aoa)PG-NH$_2$	1478.58/1477.79
		H-SHwWK(Dau=Aoa)-OH	1325.40/1324.04
		H-wWK(Dau=Aoa)-OH	1101.18/1101.01
		H-K(Dau=Aoa)-OH	728.75/728.34
		<EHWSHwW-OH	1049.12/1049.06
**K2**	[^{4}Lys(Bu), ^{8}Lys(Dau=Aoa)]	<EHWK(Bu)HDWK(Dau=Aoa)PG-NH$_2$	1953.07/1952.79
		<EHWK(Bu)HDWK(Dau=Aoa)-OH	1799.92/1799.69
		H-HDWK(Dau=Aoa)PG-NH$_2$	1320.36/1319.95
		H-HDWK(Dau=Aoa)-OH	1167.18/1166.91
		H-K(Dau=Aoa)PG-NH$_2$	881.94/881.44
		H-K(Dau=Aoa)P-OH	825.86/825.40
		H-K(Dau=Aoa)-OH	728.75/728.37
		<EHWK(Bu)HD-OH	902.96/902.84
		<EHWK(Bu)-OH	650.73/650.71
		<EHW-OH	452.46/452.31
		H-DW-OH	319.32/319.27
**4**	[^{4}Lys(Bu), ^{6}D-Asp, ^{8}Lys(Dau=Aoa)]	<EHWK(Bu)HdWK(Dau=Aoa)PG-NH$_2$	1953.07/1952.79
		<EHWK(Bu)HdWK(Dau=Aoa)P-OH	1897.03/1896.90
		<EHWK(Bu)HdWK(Dau=Aoa)-OH	1799.92/1799.81
		H-WK(Bu)HdWK(Dau=Aoa)-OH	1551.67/1551.59
		H-K(Bu)HdWK(Dau=Aoa)PG-NH$_2$	1518.65/1517.88
		H-K(Bu)HdWK(Dau=Aoa)-OH	1365.46/1364.92
		H-HdWK(Dau=Aoa)-OH	1167.18/1166.90
		<EHWK(Bu)-OH	650.73/650.43
		<EHW-OH	452.46/452.3
**5**	[^{4}Lys(Bu), ^{6}D-Glu, ^{8}Lys(Dau=Aoa)]	<EHWK(Bu)HeWK(Dau=Aoa)PG-NH$_2$	1967.13/1966.82
		<EHWK(Bu)HeWK(Dau=Aoa)P-OH	1911.06/1910.81
		<EHWK(Bu)HeWK(Dau=Aoa)-OH	1813.94/1813.70

		H-WK(Bu)HeWK(Dau=Aoa)-OH	1565.70/1565.13
		H-HeWK(Dau=Aoa)-OH	1181.22/1180.67
**5**	[^{4}Lys(Bu), ^{6}D-Glu, ^{8}Lys(Dau=Aoa)]	H-K(Bu)HeWK(Dau=Aoa)-OH	1379.49/1378.93
		<EHW-OH	452.46/452.31
**6**	[^{4}Lys(Bu), ^{6}D-Trp, ^{8}Lys(Dau=Aoa)]	<EHWK(Bu)HwWK(Dau=Aoa)PG-NH_2	2024.22/2024.25
		<EHWK(Bu)HwWK(Dau=Aoa)P-OH	1968.16/1967.84
		<EHWK(Bu)HwWK(Dau=Aoa)-OH	1871.04/1870.50
		<EHWK(Bu)HwW-OH	1160.30/1160.21
		H-K(Bu)HwWK(Dau=Aoa)-OH	1436.59/1436.24
		H-HwWK(Dau=Aoa)-OH	1238.32/1232.04
		H-K(Dau=Aoa)-OH	728.75/728.33
		<EHWK(Bu)-OH	650.73/650.43
		H-HwW-OH	527.58/527.37

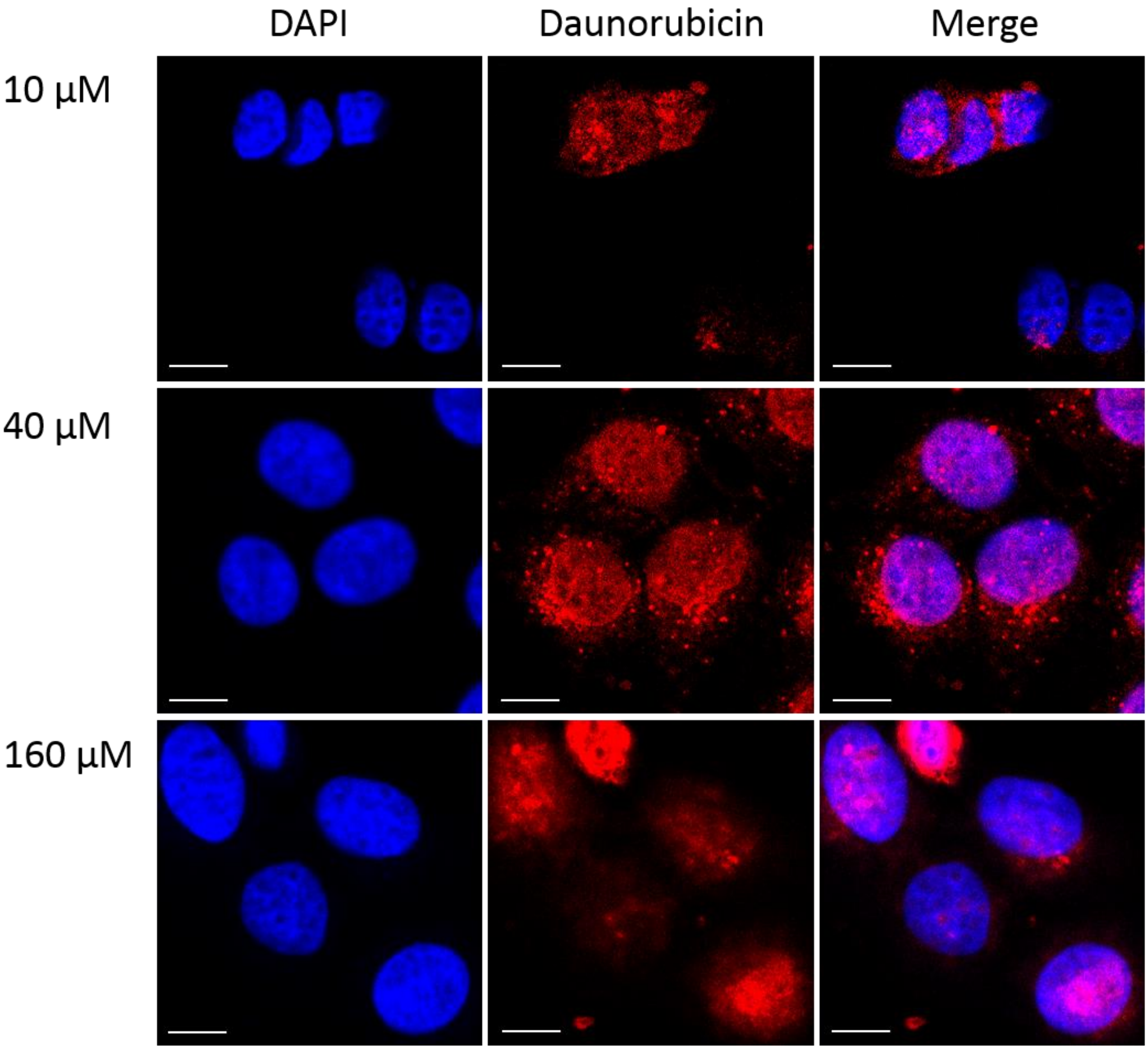

Figure S9. Cellular uptake of bioconjugate **K1** (10 µM, 40 µM and 160 µM) visualized by confocal laser scanning microscopy (CLSM) (scale bars represent 10 µm).

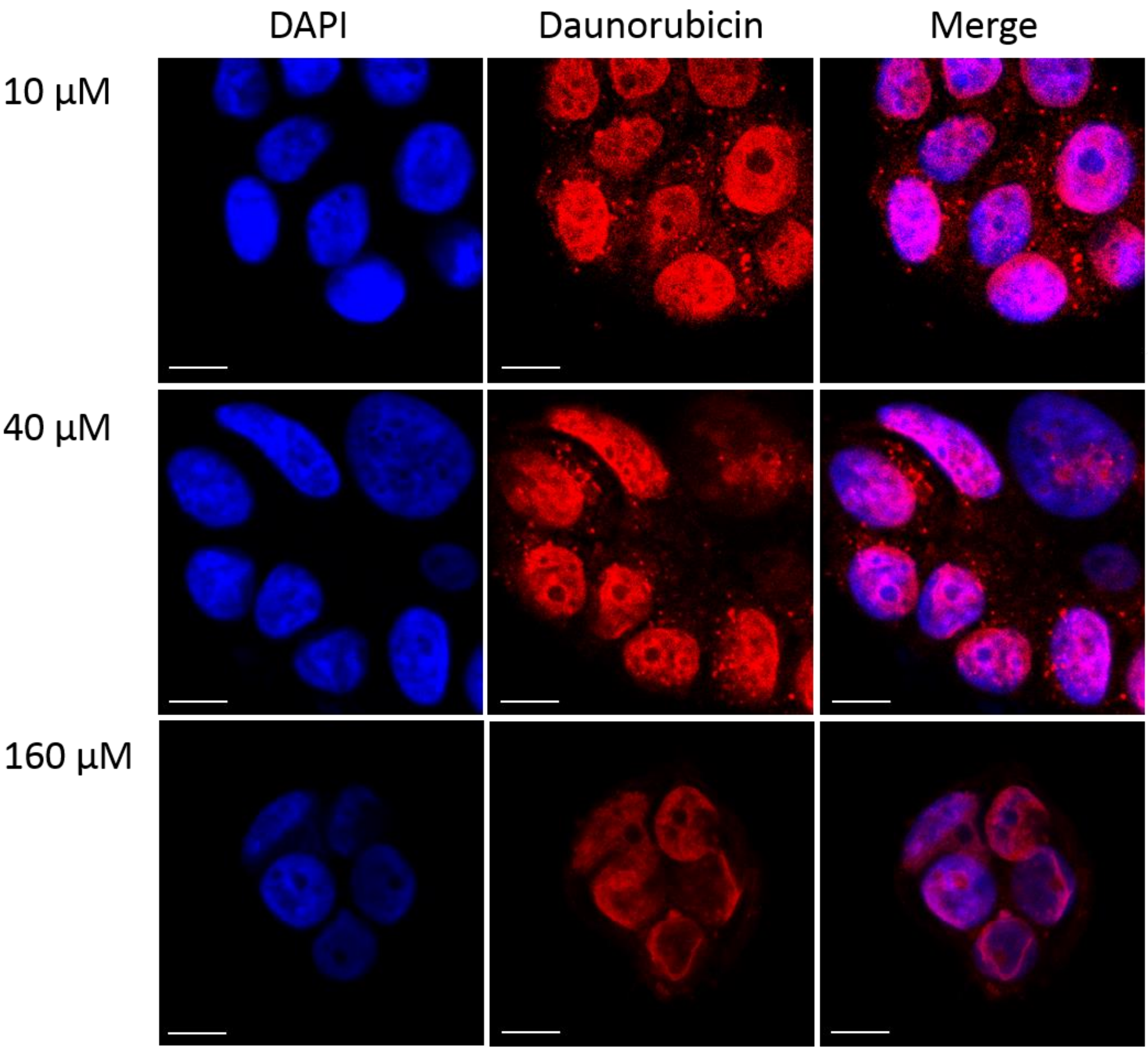

Figure S10. Cellular uptake of bioconjugate **1** (10 µM, 40 µM and 160 µM) visualized by confocal laser scanning microscopy (CLSM) (scale bars represent 10 µm).

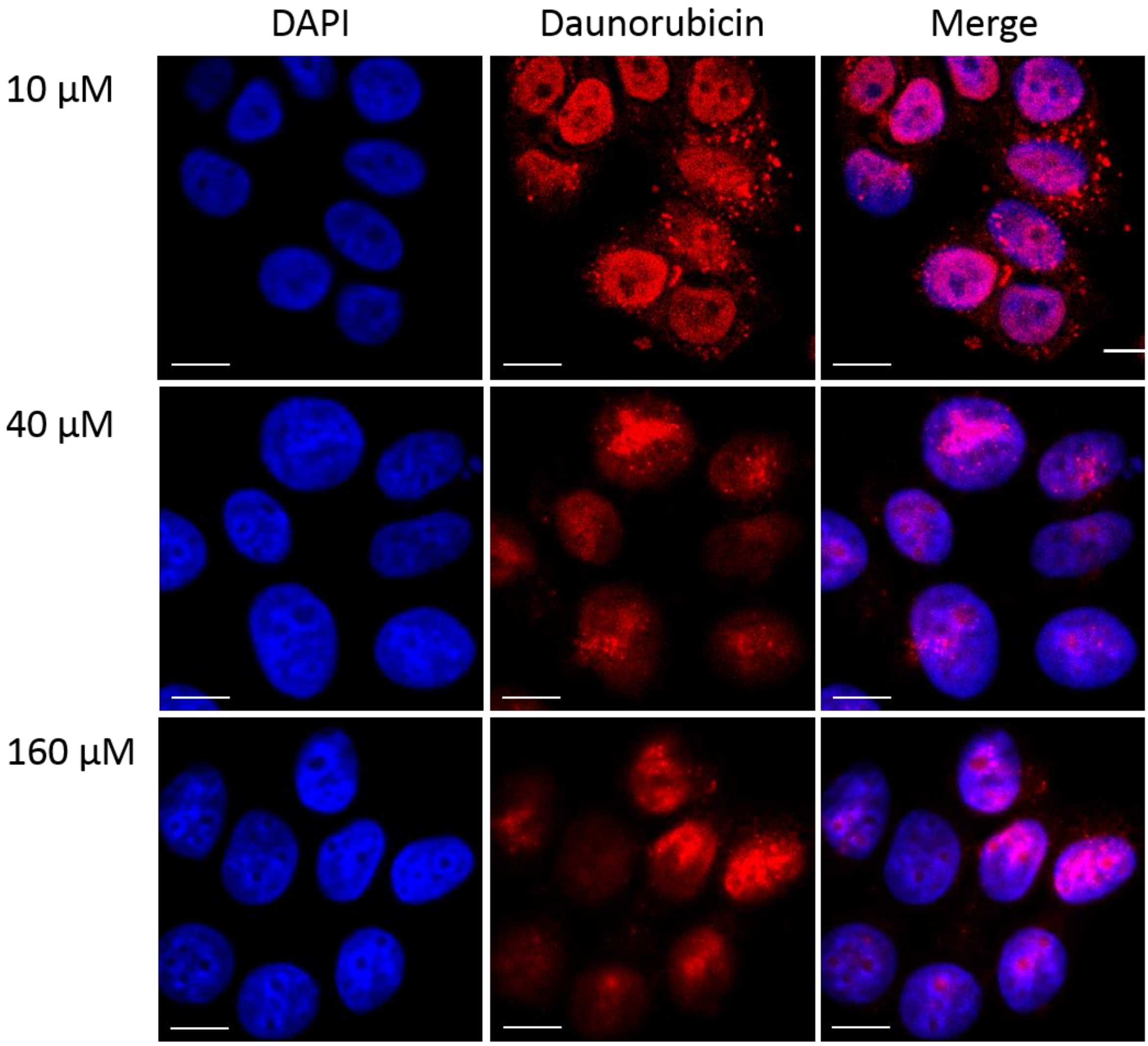

Figure S11. Cellular uptake of bioconjugate **2** (10 µM, 40 µM and 160 µM) visualized by confocal laser scanning microscopy (CLSM) (scale bars represent 10 µm).

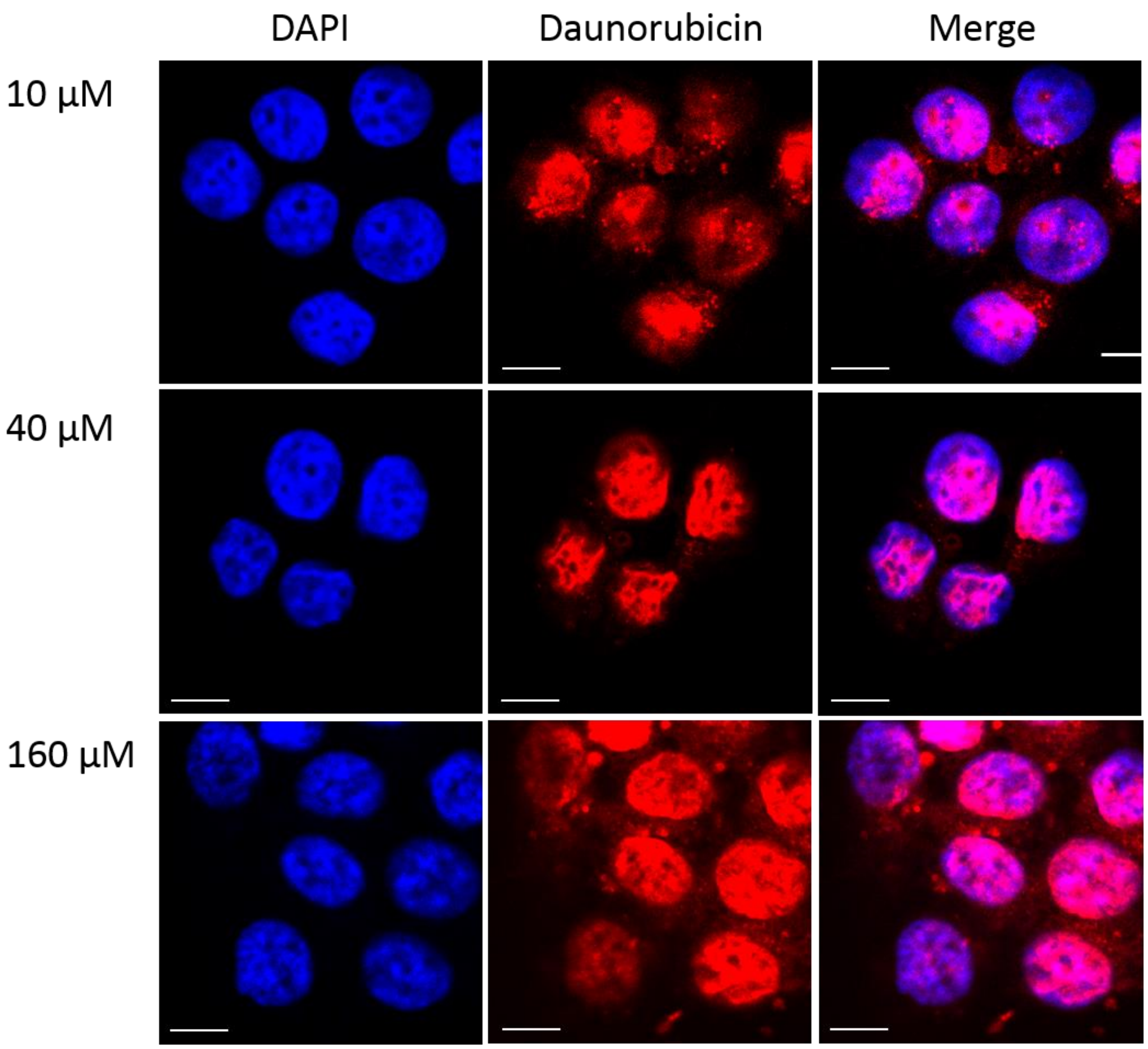

Figure S12. Cellular uptake of bioconjugate **K2** (10 µM, 40 µM and 160 µM) visualized by confocal laser scanning microscopy (CLSM) (scale bars represent 10 µm).

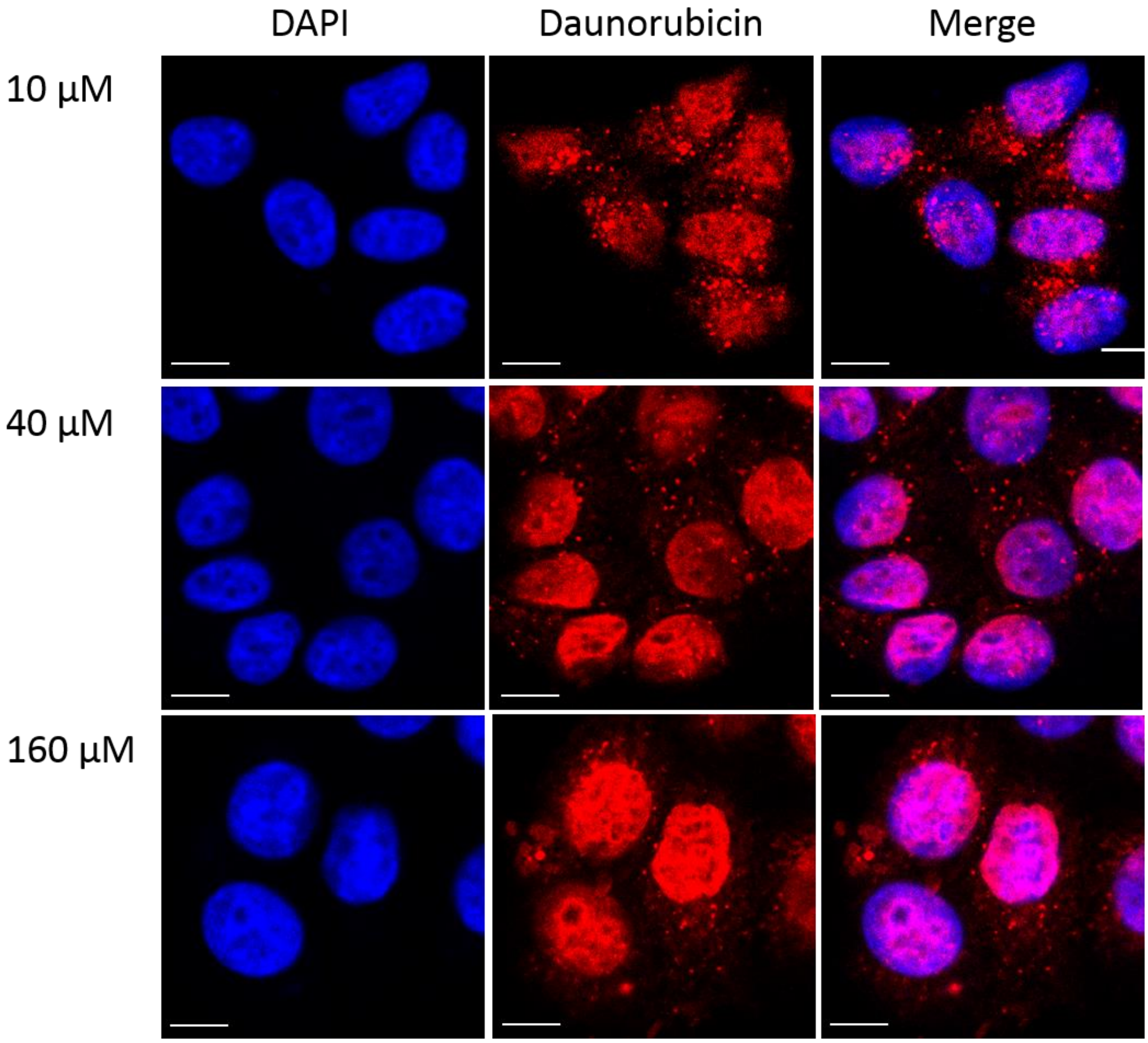

Figure S13. Cellular uptake of bioconjugate **4** (10 µM, 40 µM and 160 µM) visualized by confocal laser scanning microscopy (CLSM) (scale bars represent 10 µm).

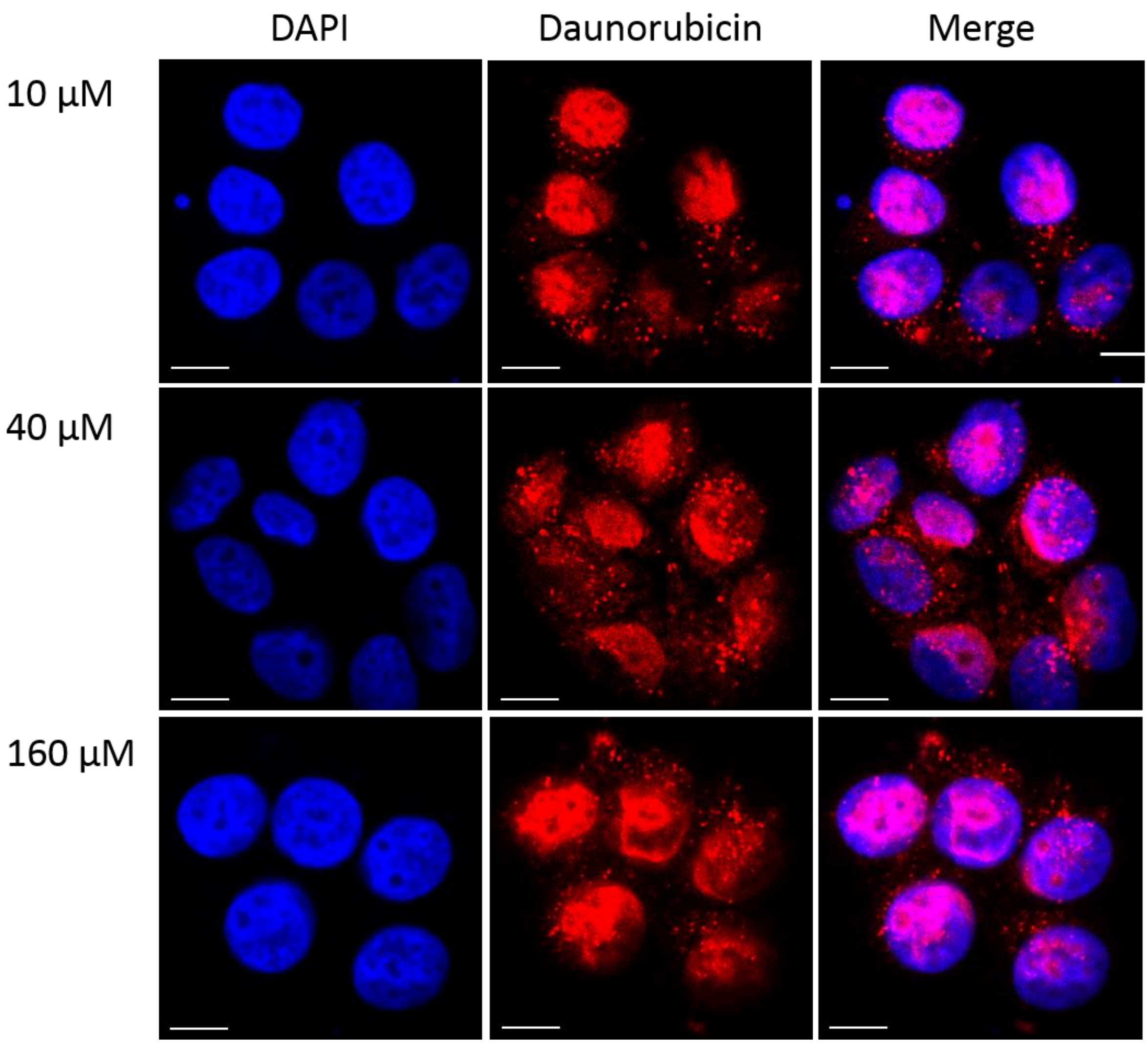

Figure S14. Cellular uptake of bioconjugate **5** (10 µM, 40 µM and 160 µM) visualized by confocal laser scanning microscopy (CLSM) (scale bars represent 10 µm).

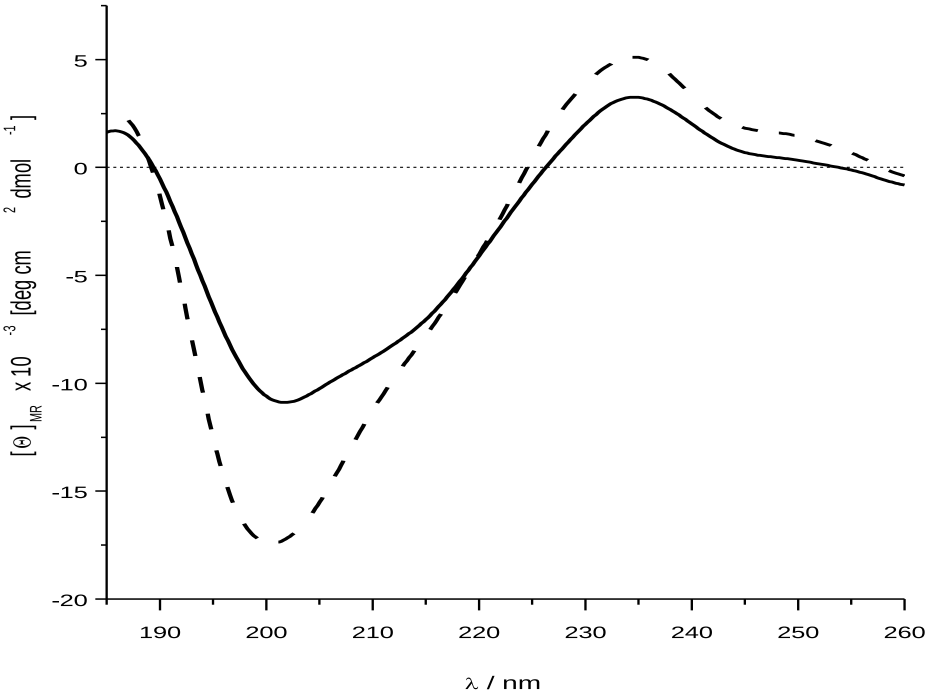

Figure S15. Far-UV ECD spectra of **K1** (dash) and **1** (solid) in water.

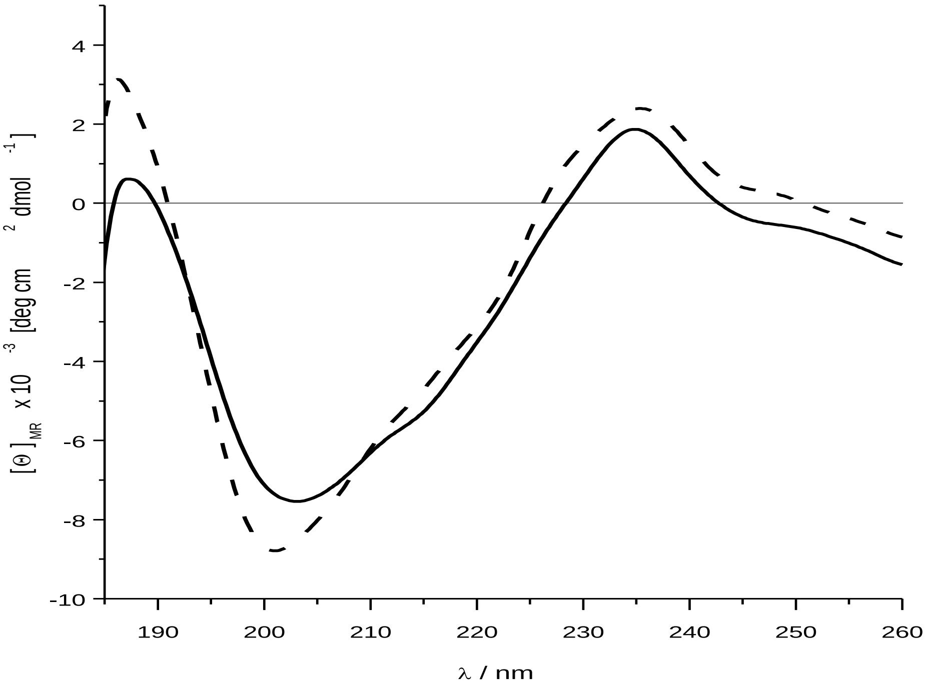

Figure S16. Far-UV ECD spectra of **K2** (dash) and **4** (solid) in water.
